# Metabolomics in aging research: aging markers from organs

**DOI:** 10.3389/fcell.2023.1198794

**Published:** 2023-06-16

**Authors:** Weicheng Fang, Shuxin Chen, Xuejiao Jin, Shenkui Liu, Xiuling Cao, Beidong Liu

**Affiliations:** ^1^ State Key Laboratory of Subtropical Silviculture, School of Forestry and Biotechnology, Zhejiang A&F University, Hangzhou, China; ^2^ Department of Chemistry and Molecular Biology, University of Gothenburg, Gothenburg, Sweden

**Keywords:** aging, metabolomics, metabolite, biomarker, aging clock

## Abstract

Metabolism plays an important role in regulating aging at several levels, and metabolic reprogramming is the main driving force of aging. Due to the different metabolic needs of different tissues, the change trend of metabolites during aging in different organs and the influence of different levels of metabolites on organ function are also different, which makes the relationship between the change of metabolite level and aging more complex. However, not all of these changes lead to aging. The development of metabonomics research has opened a door for people to understand the overall changes in the metabolic level in the aging process of organisms. The omics-based “aging clock” of organisms has been established at the level of gene, protein and epigenetic modifications, but there is still no systematic summary at the level of metabolism. Here, we reviewed the relevant research published in the last decade on aging and organ metabolomic changes, discussed several metabolites with high repetition rate, and explained their role *in vivo*, hoping to find a group of metabolites that can be used as metabolic markers of aging. This information should provide valuable information for future diagnosis or clinical intervention of aging and age-related diseases.

## Introduction

Human life expectancy has gradually increased with the development of human society. According to the World Health Organization report, the number and proportion of the population aged 60 and over is increasing. In 2019, there were 1 billion people aged 60 and over. By 2030, this number will increase to 1.4 billion, and will rise to 2.1 billion by 2050 (World Health Administration, 2020). This growth is unprecedented and will accelerate in the coming decades, especially in developing countries (Chao et al., 2021). Aging causes a decline in many bodily functions, such as decreased muscle strength, organ function, and bone density. These adverse effects make older people more susceptible to disease. The incidence of neurodegenerative diseases, such as Alzheimer’s disease (AD) and Parkinson’s disease, shows a strong correlation with age (Mattson and Arumugam, 2018). How to maintain health and physical function for longer periods of time to achieve “healthy aging” and ultimately extend “healthy lifespan” is a problem that has been pondered by humans for thousands of years. In the twentieth century, it was discovered that while the decline of cognitive and physical abilities may be an inevitable consequence of aging, changes in diet and metabolism could delay the onset of this consequence (Canevelli et al., 2016; Sanders et al., 2016). However, the exact mechanisms of aging are still unknown. Whether changes in several key pathways or substances in the body control the aging process, or whether aging is the result of all the substances in the body beginning to deteriorate and working together at the same time, still needs to be confirmed by further research.

In recent years, an increasing number of researchers have begun to use omics technology to study normal aging because of its high-throughput characteristics, such as proteomics, genomics, transcriptomics, and metabolomics. Several “aging clocks” have been established to predict the onset of aging based on relevant omics research data, such as the proteome clock, the transcriptome clock, and the epigenetic clock (Fleischer et al., 2018; Horvath and Raj, 2018; Tanaka et al., 2018; Rutledge et al., 2022). Using metabolomics platforms, researchers can perform targeted or non-targeted tracking of large numbers of metabolites simultaneously, and compare metabolomic sample data from aging subjects, allowing them to understand changes in the aging process through changes in metabolite levels. Due to the functional differences between different organs, their respective metabolite compositions will also differ. In addition, the levels of metabolites in the same organ will be different in different age groups due to the different needs of the body’s life activities. Understanding the mechanisms underlying such differences will give us the opportunity to understand the mysteries of aging.

Blood and urine samples are readily available, and the blood contains metabolites from various tissues and organs, so using blood or urine metabolites for research can reflect the overall situation of the body to some extent, and changes in the blood and urine metabolome during aging have been well described and summarized (Roberts et al., 2020; Clement et al., 2019; Johnson et al., 2019; Kondoh et al., 2020; Shao and Le, 2019; van den Akker et al., 2020; Robinson et al., 2020; Hertel et al., 2016; Adav and Wang, 2021). However, due to the fact that not all metabolites in tissues are completely excreted in blood or urine, and that changes in organ activity can have a significant impact on the metabolites in those organs, this advantage also brings with it the difficulty of determining which changes are truly reflected in the metabolomic data obtained from the blood and urine (Tryggvason and Wartiovaara, 2005; Rutledge et al., 2022). Therefore, the use of metabolomics data from specific tissues or organs can avoid the problem of metabolic signals being masked during the aging process (Schaum et al., 2020; Tabula Muris, 2020). In addition, appropriate *in vitro* cell model omics data can also represent the changes in organs during aging or disease to some extent (Chao et al., 2021).

Herein, we performed a detailed literature search for metabolic studies in multiple tissues and species related to aging (PubMed and Google Scholar search with the criteria “aging/ageing,” “senescence,” “metabolites,” “metabolome”), which consulted over a thousand related articles in total. The manuscripts were then screened and reviewed, and the inclusion criteria for a manuscript included natural aging, metabolome results published on or after 2011, publications/authors provided list of significant/all metabolites, and choose those using nature aging mice, rats, human organs or *in vitro* aging cell models as research objects. Among the articles finally selected, eight articles described changes in the metabolome of aging cell models, eight described changes in the metabolome of aging muscle, four described changes in the metabolome of aging brain, four described changes in the metabolome of aging liver, two described changes in the metabolome of aging spleen. In addition, one article each described changes in the metabolome of the aging eye, skin, kidney, lung, and testes. Of these articles, eight focused on rats, seven on mice, and eight on human tissues or cell models. The measurements were performed using multiple platforms including but not limited to liquid chromatography-mass spectrometry-based metabolome and nuclear magnetic resonance-based metabolome. Using the authors’ summary of the experimental data, we summarized more than 130 metabolites showed significant changes in the aging stage, and 47 metabolites appeared frequently (more than or equal to three articles) (Table 1). Among the 47 recurrent metabolites, 16 mostly decrease with aging in different organs, 7 mostly increase with aging. The remaining 24 metabolites showed different trends with aging in different species as well as in different tissues, and overall there did not seem to be a clear trend associated with aging. By collecting existing reports on the relationship between these metabolites and aging, we selected 14 metabolites with apparent changes in level and known to be closely related to the aging process for further discussion (Table 2). In this review, we describe the changes in metabolites during organ aging and discuss the analysis of changes in metabolites levels, as well as some metabolites that can serve as potential biomarkers of aging with apparent trends, providing potential references for future research.

**TABLE 1:** Changes of metabolites in different aging organs.

**Metabolite**	**Organ type**										**References**
	**Brain**	**Cell model**	**Eye**	**Liver**	**Muscle**	**Skin**	**Kidney**	**Lung**	**Spleen**	**Testes**	
**Carbohydrate metabolism**											
2-Hydroxyglutaric acid			↓								Wang et al. (2018)
Acetyl-CoA		↓			↓						James et al. (2015), Hoshino et al. (2022)
Aconitic acid		↑									Wu et al. (2017)
Alpha-ketoglutaric acid		↑	↓								Wu et al. (2017), Wang et al. (2018)
Citric acid		↓↑			↑						James et al. (2015), Nagineni et al. (2021), Hoshino et al. (2022)
Fructose-1,6-bisphosphate		↓↓	↓		↑↓						Garvey et al. (2014), Wu et al. (2017), Wang et al. (2018), Nagineni et al. (2021)
Fructose-6-phosphate		↑↓			↓						James et al. (2015), Wu et al. (2017), Hoshino et al. (2022)
Fumaric acid		↓↓↑		↓	↓ ↓			↓	↓		Garvey et al. (2014), Wu et al. (2017), Yi et al. (2020), Nagineni et al. (2021), Zhang et al. (2021), Zhou et al. (2021)
Glucose				↓	↑↓↓↑				↑	↓	Houtkooper et al. (2011), Garvey et al. (2014), Tepp et al. (2017), Jarak et al. (2018), Zhou et al. (2021), Hoshino et al. (2022)
Glucose-1,6-bisphosphate					↑↓						Garvey et al. (2014)
Glucose-6-phosphate		↓↓↑			↓						James et al. (2015), Wu et al. (2017), Fernandez-Rebollo et al. (2020), Hoshino et al. (2022)
Glucose-1-phosphate		↓									Fernandez-Rebollo et al. (2020)
Glyceraldehyde-3-phosphate		↓									Nagineni et al. (2021)
Glycerate-1,3-bisphosphate		↓									Nagineni et al. (2021)
Glycerate-2-phosphate		↓↓			↓						Wu et al. (2017), Kato et al. (2021), Nagineni et al. (2021)
Glycerate-3-phosphate		↓↓↑			↑↓↓						Garvey et al. (2014), James et al. (2015), Wu et al. (2017), Kato et al. (2021), Nagineni et al. (2021)
Glycogen					↑						Zhou et al. (2021)
Isocitrate		↑									Nagineni et al. (2021)
Lactate	↓	↑↑↓		↓	↓↑↓				↑		Houtkooper et al. (2011), James et al. (2015), Tepp et al. (2017), Yi et al. (2020), Nagineni et al. (2021), Zhang et al. (2021), Zhou et al. (2021)
Malic acid		↓↑	↓		↓						Garvey et al. (2014), Wu et al. (2017), Wang et al. (2018), Nagineni et al. (2021)
Malonate		↑						↓			Yi et al. (2020), Zhang et al. (2021)
Maltose				↑	↑↑						Houtkooper et al. (2011), Garvey et al. (2014)
Oxaloacetate		↑	↓								Wu et al. (2017), Wang et al. (2018)
Phosphocreatine	↓				↓↑↓						Hunsberger et al. (2020), Wilkinson et al. (2020), Kato et al. (2021), Hoshino et al. (2022)
Phosphoenolpyruvate		↑↓			↑↓↓						Garvey et al. (2014), James et al. (2015), Wu et al. (2017), Kato et al. (2021)
Pyruvate		↑↑↑	↑		↑						James et al. (2015), Wang et al. (2018), Fernandez-Rebollo et al. (2020), Nagineni et al. (2021), Hoshino et al. (2022)
Succinic acid		↓↑			↓						Wu et al. (2017), Nagineni et al. (2021), Hoshino et al. (2022)
Succinyl							↓				Zhang et al. (2021)
Succinylcarnitine					↓						Garvey et al. (2014)
**Amino Acid Metabolism**											
3-Methylhistidine					↑						Garvey et al. (2014)
Alanine		↑↓↓		↑	↑↑↓↓↓	↑					Kuehne et al. (2017), Tepp et al. (2017), Wesley et al. (2019), Yi et al. (2020), Domingo-Orti et al. (2021), Nagineni et al. (2021), Zhou et al. (2021), Zhuang et al. (2021), Hoshino et al. (2022)
Acylamino base				↑							Son et al. (2012)
Acetylcysteine			↑								Wang et al. (2018)
Anserine					↓						Garvey et al. (2014)
Arginine	↑	↓			↑↓						Hunsberger et al. (2020), Nagineni et al. (2021), Zhou et al. (2021), Hoshino et al. (2022)
Aspartate					↓↑						Zhuang et al. (2021), Hoshino et al. (2022)
Betaine				↑	↓					↓	Son et al. (2012), Jarak et al. (2018), Zhuang et al. (2021)
Carnosine					↓↓						Garvey et al. (2014), Hoshino et al. (2022)
Creatine		↓			↑↑↓↓		↑			↓	Tepp et al. (2017), Jarak et al. (2018), Yi et al. (2020), Zhang et al. (2021), Zhou et al. (2021), Hoshino et al. (2022)
Cysteine			↑↓								Wang et al. (2018)
Cysteine-Glycine			↑								Wang et al. (2018)
Dimethylarginine		↑									James et al. (2015)
Dimethylglycine		↓			↓						Morrison et al. (2019), Zhuang et al. (2021)
Gamma-aminobutyric acid	↓				↓						Zheng et al. (2016b), Zhuang et al. (2021)
Glutamate	↓	↓↓			↑↓	↑					Zheng et al. (2016b), Kuehne et al. (2017), Tepp et al. (2017), Yi et al. (2020), Nagineni et al. (2021), Zhuang et al. (2021)
Glutamine		↑↑↓			↑↓	↑					Kuehne et al. (2017), Tepp et al. (2017), Morrison et al. (2019), Yi et al. (2020), Nagineni et al. (2021), Zhuang et al. (2021)
Glutathione	↓	↓↓			↓↓						Wesley et al. (2019), Yi et al. (2020), Nagineni et al. (2021), Zhuang et al. (2021), Hoshino et al. (2022)
Glycine		↓↓			↓↓↓						Yi et al. (2020), Nagineni et al. (2021), Zhou et al. (2021), Zhuang et al. (2021), Hoshino et al. (2022)
Glycyl leucine		↓									James et al. (2015)
Glycyl valine		↓									James et al. (2015)
Hydroxyproline					↑↓						Zhuang et al. (2021), Hoshino et al. (2022)
Hypotaurine		↓	↓								Wang et al. (2018), Fernandez-Rebollo et al. (2020)
Isoleucine	↑↑	↑↓			↑↓↑	↑	↓	↑		↑	Kuehne et al. (2017), Jarak et al. (2018), Wesley et al. (2019), Yi et al. (2020), Zhang et al. (2021), Zhou et al. (2021), Zhuang et al. (2021)
Isoleucyl-glycine		↓									James et al. (2015)
Leucine	↑				↑↓↑↓		↓	↑		↑	Jarak et al. (2018), Zhang et al. (2021), Zhou et al. (2021), Zhuang et al. (2021), Hoshino et al. (2022)
Lysin					↓						Hoshino et al. (2022)
Methionine	↑				↓↓		↓	↑	↓		Zhang et al. (2021), Zhuang et al. (2021), Hoshino et al. (2022)
Methyl-histidine	↓		↑								Hunsberger et al. (2020)
N6-Trimethyl-lysine					↑						Garvey et al. (2014)
Ornithine		↑↓			↓↓						James et al. (2015), Nagineni et al. (2021), Zhuang et al. (2021), Hoshino et al. (2022)
Phenylalanine	↑				↑↓↑		↓	↑	↓	↑	Jarak et al. (2018), Zhang et al. (2021), Zhou et al. (2021), Zhuang et al. (2021), Hoshino et al. (2022)
Proline		↑↓↓			↓	↑					Kuehne et al. (2017), Yi et al. (2020), Domingo-Orti et al. (2021), Nagineni et al. (2021), Zhuang et al. (2021)
S-Adenosylmethionine					↑						Kato et al. (2021)
Serine					↓↓						Zhuang et al. (2021), Hoshino et al. (2022)
Taurine	↓	↑↓	↓		↑↓	↑					Kuehne et al. (2017), Tepp et al. (2017), Wang et al. (2018), Wesley et al. (2019), Fernandez-Rebollo et al. (2020), Yi et al. (2020), Zhuang et al. (2021)
Trimethyllysine			↑								Wang et al. (2018)
Tryptophan					↓↓						Zhuang et al. (2021), Hoshino et al. (2022)
Tyrosine	↓↑				↑↓↑↓		↓	↑	↓	↑	Jarak et al. (2018), Wesley et al. (2019), Zhang et al. (2021), Zhou et al. (2021), Zhuang et al. (2021), Hoshino et al. (2022)
Valine	↑	↓			↑↓↓	↑	↓	↑		↑	Kuehne et al. (2017), Jarak et al. (2018), Yi et al. (2020), Zhang et al. (2021), Zhuang et al. (2021), Hoshino et al. (2022)
Valyl Aspartate		↓									James et al. (2015)
Valyl glycine		↓									James et al. (2015)
**Lipid metabolism**											
3-Ureidopropionate		↑									James et al. (2015)
Acetylcarnitine					↓						Zhuang et al. (2021)
Acylcarnitine	↓				↓						Garvey et al. (2014), Zheng et al. (2016b)
Carnitine				↑	↓↓						Son et al. (2012), Garvey et al. (2014)
Cholesterol			↓	↑							Son et al. (2012), Wang et al. (2018)
Choline					↑↓		↓			↑	Jarak et al. (2018), Kato et al. (2021), Zhang et al. (2021), Zhuang et al. (2021)
Dihomolinolenic acid					↓						Garvey et al. (2014)
Fatty acid			↓		↑↓↓						Garvey et al. (2014), Wang et al. (2018), Zhang et al. (2021)
Glycerol-3-phosphate				↑	↑↓						Son et al. (2012), Garvey et al. (2014), Hoshino et al. (2022)
Glycerin		↓		↓	↑						Garvey et al. (2014), James et al. (2015), Zhang et al. (2021)
Glycerophosphocholine					↑						Garvey et al. (2014)
Glycerophospholipids					↓						Garvey et al. (2014)
Inositol		↓									Yi et al. (2020)
Linoleic acid				↓	↓						Son et al. (2012), Garvey et al. (2014)
Lysophosphatidylcholine	↑				↑↓						Zheng et al. (2016b), Wilkinson et al. (2020), Zhang et al. (2021)
Oleic acid					↑						Garvey et al. (2014)
Palmitoleate					↑						Garvey et al. (2014)
Phospholipids			↓								Wang et al. (2018)
Stearic acid					↓						Garvey et al. (2014)
Steroid			↓								Wang et al. (2018)
**Nucleotide metabolism**											
7-Methylguanine		↑									James et al. (2015)
Adenine triphosphate ribonucleotide		↓↓↓			↓↓						Morrison et al. (2019), Yi et al. (2020), Kato et al. (2021), Nagineni et al. (2021), Hoshino et al. (2022)
Adenosine monophosphate					↓						Zhuang et al. (2021)
Allantoin	↓				↓		↑	↑	↑		Zhang et al. (2021), Zhuang et al. (2021)
Deoxyribose			↑								Wang et al. (2018)
Flavin adenine dinucleotide					↓						Garvey et al. (2014)
Guanine diphosphate ribonucleotide		↑		↑	↑↑						Morrison et al. (2019), Kato et al. (2021), Zhang et al. (2021), Hoshino et al. (2022)
Guanine monophosphate ribonucleotide					↑↑						Kato et al. (2021), Hoshino et al. (2022)
Hypoxanthine		↑↑		↑	↑↓						James et al. (2015), Morrison et al. (2019), Kato et al. (2021), Zhang et al. (2021), Zhuang et al. (2021)
Inosine	↓	↑		↓	↓↑↓		↓				Morrison et al. (2019), Zhang et al. (2021), Zhou et al. (2021), Hoshino et al. (2022)
Inosine monophosphate		↑		↑	↑↑						Morrison et al. (2019), Kato et al. (2021), Zhang et al. (2021), Hoshino et al. (2022)
Phosphoribosyl pyrophosphate		↓									Nagineni et al. (2021)
Ribose		↓			↓↑						Garvey et al. (2014), Nagineni et al. (2021)
Ribulose-5-phosphate					↓↑						Garvey et al. (2014)
Thymidine		↓									James et al. (2015)
Uracil	↑	↑			↑↑		↑				Morrison et al. (2019), Kato et al. (2021), Zhang et al. (2021)
Urate		↑									James et al. (2015)
Uridine	↓			↑↓	↓		↓				Son et al. (2012), Morrison et al. (2019), Zhang et al. (2021)
Xylulose-5-phosphate					↓↑						Garvey et al. (2014)
**Polyamine and NAD^+^ metabolism**											
Putrescine		↓			↓						Nagineni et al. (2021), Hoshino et al. (2022)
Spermidine					↑↓↓						Kato et al. (2021), Zhuang et al. (2021), Hoshino et al. (2022)
Spermine		↑			↑						Wilkinson et al. (2020), Nagineni et al. (2021)
Nicotinamide			↑	↓	↑		↓	↓			Wang et al. (2018), Zhang et al. (2021), Zhuang et al. (2021)
Nicotinamide adenine dinucleotide	↓	↓↓	↑	↓	↑↓↓						Garvey et al. (2014), Wang et al. (2018), Wesley et al. (2019), Yi et al. (2020), Nagineni et al. (2021), Zhuang et al. (2021), Hoshino et al. (2022)
Nicotinamide ribonucleotide		↓									Fernandez-Rebollo et al. (2020)
Nicotinamide riboside		↓									Fernandez-Rebollo et al. (2020)
Nicotinic acid	↑										Wesley et al. (2019)
Reduced nicotinamide adenine dinucleotide		↓↓↓	↓	↑							James et al. (2015), Wesley et al. (2019), Yi et al. (2020), Nagineni et al. (2021), Hoshino et al. (2022)
Reduced nicotinamide adenine dinucleotide phosphate		↓			↑						Wu et al. (2017), Hoshino et al. (2022)
**Other**											
Acetate	↑										Zhang et al. (2021)
Ethanolamine							↓				Zhang et al. (2021)
Fenugreek			↓								Wang et al. (2018)
Formate				↓							Wesley et al. (2019)
Glyceric acid			↑								Wang et al. (2018)
Indole-3-acetic acid			↓								Wang et al. (2018)
Methyl glutamate			↑								Wang et al. (2018)
Methyl-histamine	↓										Hunsberger et al. (2020)
Phosphocholine									↓		Wang et al. (2018)
Riboflavin			↑								Wang et al. (2018)
Sorbitol					↑↓						Garvey et al. (2014), Zhuang et al. (2021)
Thiamine			↓								Wang et al. (2018)

A single arrow is used to represent the research result of an article. Up arrows indicate the metabolite level increases in older organ samples compared to younger samples, and the down arrows indicate that it decreases. Red arrows represents rat tissue, green represents mouse tissue or cell model, and blue represents human tissue or cell model.

**TABLE 2:** Changes in metabolite levels with apparent trends in different aging organs and cell models.

Metabolite	Organ type		References
	Up	Down	
Adenosine triphosphate		Mice master muscle; Human Colon cancer cells; Umbilical vein endothelial cells	Morrison et al. (2019), Yi et al. (2020), Kato et al. (2021), Nagineni et al. (2021)
Fatty acids	Rat Gastrocnemius	Mice heart; eye; Rat soleus	Garvey et al. (2014), Wang et al. (2018), Zhang et al. (2021)
Glucose	Mice spleen; Rat Gastrocnemius	Mice liver, muscle; Rat Soleus, Testis	Houtkooper et al. (2011), Garvey et al. (2014), Jarak et al. (2018), Zhou et al. (2021)
Glutamate	Rat heart; Human skin	Rat brain, muscle; Human Colon cancer cells; Umbilical vein endothelial cells	Kuehne et al. (2017), Tepp et al. (2017), Wesley et al. (2019), Yi et al. (2020), Nagineni et al. (2021), Zhuang et al. (2021)
Glutamine	Human Umbilical vein endothelial cells, skin; Rat heart	Rat muscle; Human Colon cancer cells	Kuehne et al. (2017), Tepp et al. (2017), Morrison et al. (2019), Yi et al. (2020), Nagineni et al. (2021), Zhuang et al. (2021)
Glutathione		Rat brain, muscle; Human Colon cancer cells; Umbilical vein endothelial cells	Morrison et al. (2019), Wesley et al. (2019), Yi et al. (2020), Nagineni et al. (2021), Zhuang et al. (2021), Hoshino et al. (2022)
Glycine		Rat muscle; Mice muscle; Human Colon cancer cells; Umbilical vein endothelial cells	Yi et al. (2020), Nagineni et al. (2021), Zhou et al. (2021), Zhuang et al. (2021), Hoshino et al. (2022)
Isoleucine	Mice Lung, brain, heart, muscle; Rat testis, brain; Human skin	Mice kidney; Rat muscle; Human Umbilical vein endothelial cells	Kuehne et al. (2017), Jarak et al. (2018), Wesley et al. (2019), Yi et al. (2020), Zhang et al. (2021), Zhou et al. (2021), Zhuang et al. (2021)
Lactate	Mice spleen; Rat heart; Human Colon cancer cells, Fibroblast	Mice muscle, liver, brain; Human Umbilical vein endothelial cells	Houtkooper et al. (2011), James et al. (2015), Tepp et al. (2017), Yi et al. (2020), Nagineni et al. (2021), Zhang et al. (2021), Zhou et al. (2021)
Lysophosphatidylcholine	Rat brain; Human Vastus lateralis	Mice heart	Wesley et al. (2019), Wilkinson et al. (2020), Zhang et al. (2021)
Nicotinamide adenine dinucleotide	Mice eye; Rat muscle	Rat brain, liver, muscle; Human colon cancer cells; Umbilical vein endothelial cells	Garvey et al. (2014), Wang et al. (2018), Wesley et al. (2019), Yi et al. (2020), Nagineni et al. (2021), Zhuang et al. (2021), Hoshino et al. (2022)
Pyruvate	Mice eye; Human Colon cancer cells, Mesenchymal cells		Wang et al. (2018), Fernandez-Rebollo et al. (2020), Nagineni et al. (2021)
Uracil	Mice brain, heart, kidney, red blood cells, master muscle		Morrison et al. (2019), Kato et al. (2021), Zhang et al. (2021)
Valine	Mice lung, brain, heart; Rat testis; Human skin	Mice kidney; Rat muscle; Human Umbilical vein endothelial cells	Kuehne et al. (2017), Jarak et al. (2018), Yi et al. (2020), Zhang et al. (2021), Zhuang et al. (2021), Hoshino et al. (2022)

### Metabolites from carbohydrate metabolism is one of the extensively studied group as markers during aging

Carbohydrate metabolism refers to a series of complex chemical reactions of glucose (Glu), glycogen, etc. In the body. The major metabolic pathways of Glu *in vivo* include glycolysis, the tricarboxylic acid (TCA) cycle, and the pentose phosphate (PPP) pathway. The entire reaction process of glycolysis is completed in the cytoplasm, starting with the Glu, which is catalyzed by a series of enzymes to form the end-product pyruvate. Pyruvate is then reduced to lactate under anaerobic conditions. Under aerobic conditions, pyruvate enters the mitochondria and is further oxidized to produce acetyl coenzyme A (acetyl-CoA) for further oxidation in the TCA cycle, which is the primary pathway for energy production in the organism. In addition, the PPP is another important pathway for Glu oxidation and catabolism, and is designed to provide some of the raw materials needed for biosynthesis, rather than for cellular energy supply (Anastasiou et al., 2011; Gruning et al., 2011).

In the testis of aging rats, Glu levels are decreased (Jarak et al., 2018), and the masseter muscle showed lower levels of 3-phosphoglycerate, 2-phosphoglycerate, and phosphoenolpyruvate (PEP) (Kato et al., 2021). In aged mice, pyruvate levels are elevated in the eye, but fructose-1,6-bisphosphate (FBP) showed different changes in different parts of the eye (Wang et al., 2018). In cellular models, the trend in glycolysis consistent with the trend in rats and mice (Wu et al., 2017; Nagineni et al., 2021), suggesting that the degree of glycolysis is upregulated in senescent cells (Wu et al., 2017). In addition, the PPP also shows an increased trend (Garvey et al., 2014), while the TCA cycle shows the opposite trend. In the skeletal muscle of aging rats, the levels of fumaric acid and malic acid, intermediate metabolites of the TCA cycle, were decreased in older individuals. Levels of succinylcarnitine and flavin adenine dinucleotide (FAD) were also reduced (Garvey et al., 2014). In addition, the liver, spleen, lungs, and eye of aging mice all have decreased levels of fumaric acid, oxaloacetate, malic acid, and α-ketoglutarate (Wang et al., 2018; Zhang et al., 2021). Studies at the cell level yielded similar results. Senescent cells showed a downward trend in malic acid, fumaric acid, and succinic acid levels, while isocitrate and citric acid levels were relatively elevated (Yi et al., 2020; Nagineni et al., 2021). In addition to the major metabolites listed above, other metabolic intermediates such as allantoin, acetate, ethanolamine, choline, phosphorylcholine, 2-hydroxyglutarate, succinyl, malonic acid, and citrate, also change with age (Table 1).

Because of the different functions of organs in different parts of the body, their metabolite level changes at the same time can be distinguished. For example, in rat gastrocnemius muscle, the levels of glycolytic intermediates such as FBP, and PEP are significantly increased, but in soleus muscle, FBP, PEP, glucose-6-phosphate (G6P), etc., showed the opposite changes in the same age group of rats (Garvey et al., 2014). Studies have shown that the aging process of the soleus muscle may be later than that of the gastrocnemius muscle (Oliveira et al., 2019; Yanar et al., 2019), which may account for the different metabolic trends seen in these two muscles in rats of the same age. In addition, relatively high levels of the TCA cycle intermediate fumaric acid, malic acid, succinic acid, and α-ketoglutarate were detected in human breast cancer cells with a senescent phenotype induced by doxorubicin (Wu et al., 2017). These changes are different from the aforementioned changes in mitochondria-related metabolome in normal aging cells. The reason for this phenomenon may be that when the damage accumulation does not reach the threshold, cells can repair the damaged DNA by increasing the strength of the PPP and TCA pathways to synthesize nucleotides, thereby compensating for the cellular dysfunctions caused by DNA damage as soon as possible (Gewirtz, 1999; Thorn et al., 2011).

Pyruvate is the key metabolite linking glycolysis and the TCA cycle. Pyruvate has been shown to have antioxidant, anti-nitrite stress, and anti-inflammatory effects, among others (Flaherty et al., 2010; James et al., 2015). In addition, pyruvate can undergo transamination reactions with glutamate to produce alanine and α-ketoglutarate, which are used as reaction intermediates to participate in the remaining steps of the TCA cycle. A study has shown that the consumption priority of pyruvate is higher than that of glucose in isolated mouse skeletal muscle (Khattri et al., 2022). In addition, pyruvate can counteract hypoxic lactic acidosis and the Warburg effect by increasing the ratio of nicotinamide adenine dinucleotide to reduced nicotinamide adenine dinucleotide (NAD^+^/NADH) (Hu et al., 2013). It also protects the structural integrity of the mitochondria and the function of the endoplasmic reticulum, thereby preventing cell apoptosis (Zhang et al., 2020a; Li et al., 2020). In addition, studies have shown that artificially elevated pyruvate can increase NAD^+^ levels (Iannetti et al., 2018; Kim et al., 2018), and there are no reports of adverse effects, which has led researchers to consider the possibility of using pyruvate or pyruvate derivatives as drugs to treat certain diseases. Some scientists believe that pyruvate can be used instead of NAD^+^ as an exogenous supplement to treat diabetes, but more research is needed to verify this hypothesis (Zhang et al., 2020b; Zhou, 2021). Thus, the elevated levels of pyruvate, a key central metabolite, during the aging phase, indicate that the downstream pathways it mediates are inhibited. Given the variety of stress conditions that occur during aging, inhibition of these pathways is likely to be a direct manifestation of aging. All of these results suggest that pyruvate can be used as an indicator of organ aging.

In addition to pyruvate, Glu is one of our first choices as a marker metabolite of aging, but Glu in different organs does not change consistently with age. Studies have shown that the Glu uptake capacity of high-energy-consuming organs such as the brain and skeletal muscle is reduced due to the decline in insulin signaling, insulin sensitivity, and Glu transporter levels that occurs with aging (Muzumdar et al., 2004; Karakelides et al., 2010; dos Santos et al., 2012). The expression levels of phosphoenolpyruvate carboxykinase (PEPCK) and Glu-6-phosphatase (G6Pase) were increased in the livers of aging rats, which ultimately increased hepatic Glu production (Gaspar et al., 2020). These results suggest that different organs have different levels of Glu requirements, which means that Glu may not be a suitable aging marker for the whole-body. However, because of its high detection sensitivity, it is possible to combine changes in Glu levels with other metabolites in specific organs to provide a targeted indication of organ age.

Lactate is reduced from pyruvate under anaerobic conditions, and several studies in different organs have found that lactate levels change with age as well as in senescent cell models (Houtkooper et al., 2011; Tepp et al., 2017; Nagineni et al., 2021; Zhang et al., 2021), but the of change trends are different. Lactate has long been considered a metabolic waste product for a long time. However, studies have shown that most of the lactate produced by muscle contraction is taken up by other organs through the bloodstream and used as fuel for oxidation to produce energy, a phenomenon now known as the lactate shuttle (Brooks, 1986; Bergersen, 2007; Adeva-Andany et al., 2014). Central nervous system lactate increase as plasma lactate levels increase, with decreased Glu uptake, suggesting that the brain may prefer lactate to Glu as a raw material for energy production (Smith et al., 2003). Muscle cells can convert lactate to pyruvate, which can enter the TCA cycle directly, if oxygen is available, and the liver can also convert lactate to Glu through gluconeogenesis (Bergman et al., 2000; Meyer et al., 2002; Le et al., 2010; Emhoff et al., 2013). In fact, lactate can not only directly enter the mitochondria directly to complete the oxidation process to pyruvate, but it can also be converted to pyruvate in the cytoplasm and then transported to the mitochondria, and an increase in lactate intake can help cells generate more energy faster through the TCA process (Bouzat et al., 2014). In recent years, studies have shown that lactate can bind to GPR81 (also known as hydroxycarboxylic acid receptor HCAR1), a member of the members of the G protein-coupled receptor family (Lauritzen et al., 2014), shedding new light on how lactate is involved in more complex signaling processes (Bozzo et al., 2013). Overall, similar to Glu, the general changes in lactate levels in aging organs suggest that lactate is closely related to aging and could be a targeted indicator of organ age.

**FIGURE 1:**
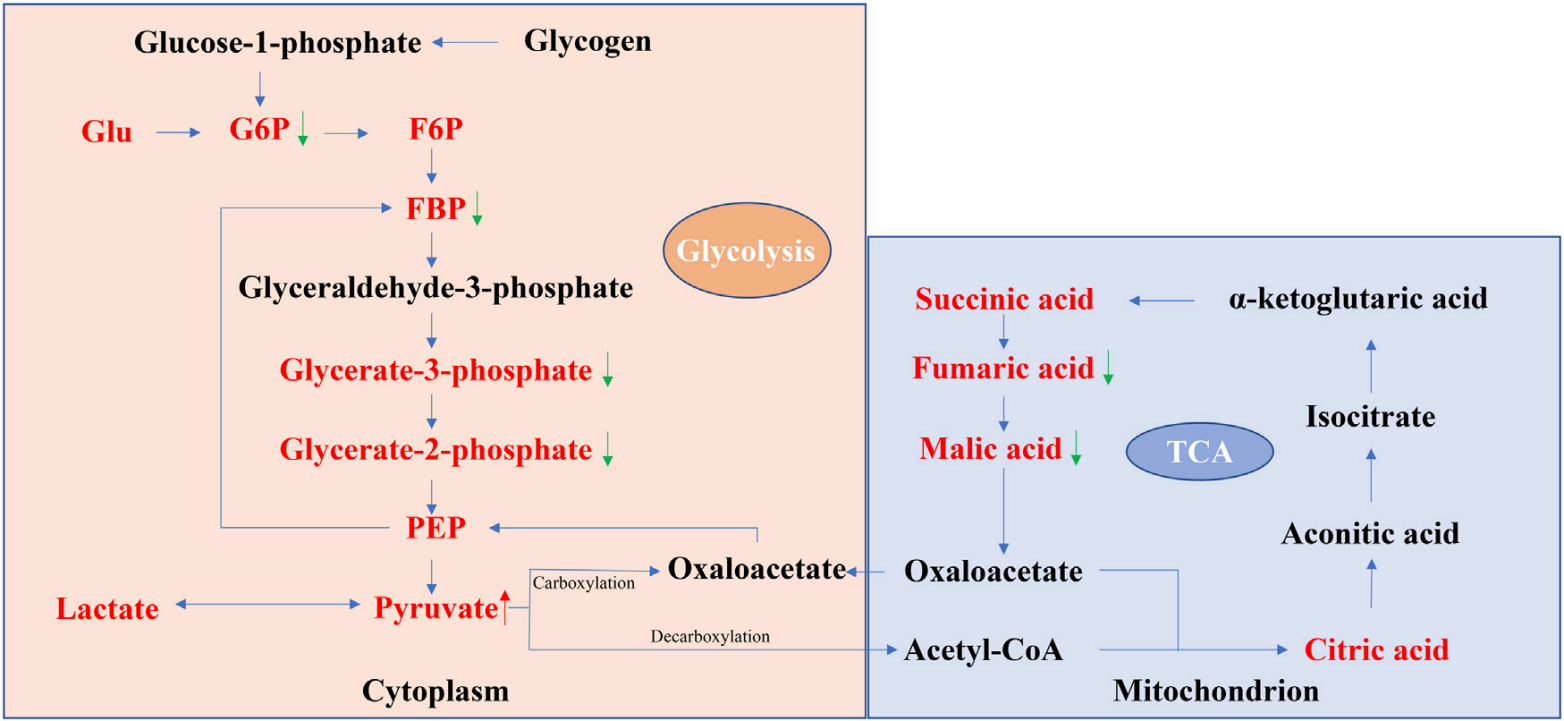
Schematic representation of glycolysis and the TCA cycle. The red font indicates the metabolites with significant changes in the level of aging organs found in most studies (data from Table 1). The red up arrow indicates that the changes of metabolites in different reports and tissues are mostly increasing, while the green down arrow indicates the opposite. In aging organs, the levels of the glycolytic intermediate products G6P, FBP, glycorate-3-phosphate, and glycorate-2-phosphate and the TCA intermediate products fumaric acid and malic acid are relatively low, while the levels of the glycolytic end product pyruvate are relatively high, reflecting that the glycolytic flux increased at the stage of organ aging, while the TCA cycle flux decreased.

Taken together, these results suggest that the energy centers of the cell undergo a shift from the mitochondria to the cytoplasm during senescence. Changes in the levels of several metabolites reflect this age-related transition (Figure 1), such as the levels of fumaric acid and malic acid were relatively decreased in the aging stage, accompanied by a relative increase in the level of pyruvate, indicating an enhancement of the glycolytic pathway and the weaking of the TCA pathway during the aging phase. On the other hand, some modalities thought to prolong the lifespan, such as energy restriction (CR), can increase the metabolic level of the TCA cycle, which laterally reflects that the decrease in the TCA cycle may be a symbol of aging (Heilbronn and Ravussin, 2003; Mitchell et al., 2016). Based on studies summarized above, it seems that pyruvate can be used as a marker for carbohydrate metabolism changes during aging, while more research on Glu, lactate is needed to better understand their role in aging.

### Amino acid metabolites showing different trends during aging

**FIGURE 2:**
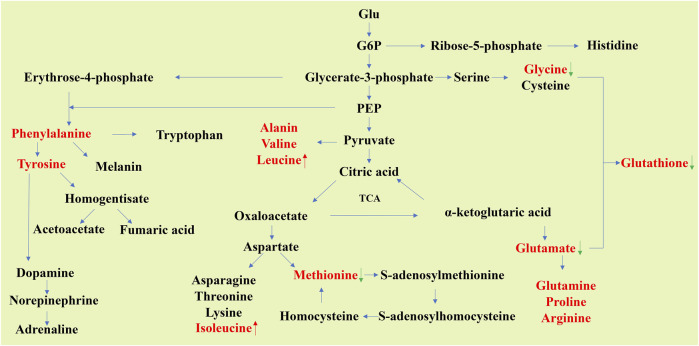
Schematic of carbon skeleton sources during nonessential amino acid synthesis. The metabolites reported in many articles that their levels in aging organs will change significantly are shown in red font (data from Table 1). The red up arrow indicates that the metabolites are increased in aging tissues, while the green down arrow indicates that they are decreased in aging tissues. The levels of glycine, glutamate, and methionine can be seen to decline in aging organs, while the opposite is true for leucine and isoleucine.

Amino acids and small-molecule peptides are also metabolites that appear frequently in many studies. Amino acids can be used to synthesize proteins necessary for life activities, and are associated with a number of other metabolites (Figure 2), and some important anti-stress substances in the body are also synthesized through amino acid metabolism, such as glutathione (GSH) and creatine (Hopkins, 1929; Kendall et al., 1930; Van Pilsum et al., 1972).

Most protein amino acids show different metabolic trends in different aging organs or cells. For example, in aged rats, glutamate level decreased while alanine, and glutamine levels increased (Table 1). In senescent human umbilical vein endothelial cells, the levels of glutamate and alanine decreased as the number of cell passages increased. In human skin samples, the level of glutamate was higher in older samples, as were the levels of glutamine and alanine (Kuehne et al., 2017). Some amino acids even show inconsistent metabolic trends in different parts of the same organ, such as cysteine. Cysteine levels increased with age in the retina and lens, but decreased in the cornea (Wang et al., 2018). The same is true for non-protein amino acids and modified amino acids, such as taurine, methylhistidine, and methylhistamine, as well as small molecular weight peptides, such as creatine (Table 1).

Glutamate is a free amino acid that is abundant in the human body and was originally known for its unique umami taste. Glutamate is an excitatory neurotransmitter (Burger et al., 1989; Watkins and Jane, 2006; Zhou and Danbolt, 2014), and because of the close link between glutamate recycling at synapses and energy metabolism (Sibson et al., 1998; Yu et al., 2018), maintaining of glutamate homeostasis at synaptic sites is of great importance for normal brain function. In addition, neurons actively take up glutamate and use it as a substrate for energy production (Sonnewald et al., 1996; Olstad et al., 2007). An increasing number of studies have shown that brain energy metabolism homeostasis is closely related to neurological diseases. Some neurological diseases are associated with abnormal glutamate levels, such as AD. Significantly lower glutamate levels have been found in patients with major depression and AD compared to healthy subjects (Rupsingh et al., 2011; Gueli and Taibi, 2013; Inoshita et al., 2018). This information raised the question of whether abnormal glutamate metabolism may be a major cause and an important manifestation of neurodegenerative diseases (Cunnane et al., 2020). Studies have shown that decreased expression of a key glutamate receptor, GLT-1, in both AD and Huntington’s disease (HD) patients and related rodent disease models reduces the efficiency of glutamate uptake by astrocytes for release into the synaptic cleft (Andersen et al., 2021). Glutamate is also active in peripheral organs such as the digestive tract, pancreas, and bone (Julio-Pieper et al., 2011; Tremolizzo et al., 2012). It has been reported that approximately 35% of the total energy consumption of intestinal mucosal cells comes from dietary glutamate (Uneyama et al., 2017). Together with the decreasing trend of glutamate in the aging rat brain described above, these results together raise the possibility of using glutamate as a marker of brain aging.

Glutamine is listed as a non-essential amino acid because it can be synthesized *in vivo*, and it is involved in many metabolic pathways as well as glutamate transfer processes in the nervous system. One article suggested that inhibiting the normal breakdown of glutamine may promote various age-related diseases by prolonging the survival time of senescent cells (Johmura et al., 2021). This is similar to the conclusion that timely removal of senescent cells in some age-related diseases can maintain overall organ or body health (Xu et al., 2018; Justice et al., 2019). Here, relatively high levels of glutamine were found in several aging organs and cell models (Table 1), as if glutamine degradation in inhibited during aging. However, not all aging organs show higher glutamine levels (Table 1). Recently, a study has shown that aging mesenchymal stem cells exhibit metabolic remodeling, characterized by reduced glucose uptake, which in turn compensates for energy generation gaps by degrading glutamine (Choudhury et al., 2022). Therefore, further studies on the metabolic changes in glutamine in aging are needed.

GSH is a tripeptide synthesized from three amino acids by the enzymatic system and is a common antioxidant in cells. Although its primary anabolic sites are the liver and kidney, GSH is present in almost all cells of the body (Jana et al., 2021). Previous studies have mentioned that its levels decrease during aging (Mitchell et al., 2000; Rebrin and Sohal, 2008; Cantor and Sabatini, 2012; Homma and Fujii, 2015; Verdin, 2015; DeBerardinis and Chandel, 2016; Pavlova and Thompson, 2016), which is consistent with the results of metabolomic studies in recent years (Table 2). The oxidized form of GSH is called GSSG. GSH is the major form that exerts its function, and GSSG must be reduced to obtain GSH before the next round of reaction. GSH has significant effects in enhancing immunity and detoxification, and it has been reported in recent years that the intracellular detoxification process involving GSH has a certain delaying effect on senile deafness (Escartin et al., 2011; Scire et al., 2019; Ferreira et al., 2021; Someya and Kim, 2021). Combined with its functions, especially its antioxidant capacity, proper supplementation of GSH may have unexpected effects on aging or some age-related diseases. However, the strong antioxidant capacity of GSH itself also indicates that it is easily oxidized and has poor stability. In addition, as a tripeptide, it cannot cross the cell membrane directly by itself. These factors make it difficult to preserve and directly replenish GSH. However, the nanodrug delivery system developed in recent years may provide a new direction for artificial GSH supplementation (Li et al., 2021). According to the metabolomic research literature discussed above, GSH levels or GSH/GSSG levels in aging organs were relatively decreased in the aging stage, which means that it may indicate the aging process and could be an aging marker.

Glycine levels showed a tendency to decrease with aging (Table 1). Glycine is one of the amino acids that synthesize GSH, and its level is affected to some extent by the level of GSH, then influence the progress of aging (Kumar et al., 2020; Kumar et al., 2021). Glycine is also simultaneously the input amino acid for one-carbon metabolism and is able to contribute single carbon units to the folate cycle to produce a variety of one-carbon bound tetrahydrofolates (THF) (Locasale, 2013). These act as coenzymes in methylation reactions, including the generation of methionine by methionine synthase (METR-1 in *C. elegans*) and the universal methyl donor S-adenosylmethionine (SAMe) by S-adenosylmethionine synthase (SAMS-1) in *C. elegans* (Locasale, 2013). These output metabolites of one carbon metabolism support a number of biological functions (Locasale, 2013). In *C. elegans*, mutations in the metabolic gene SAMS-1 and levels of SAMe and S-adenosylhomocysteine (SAH) have been implicated in the regulation of senescence (Hansen et al., 2005; Cabreiro et al., 2013). Another study in mouse pluripotent stem cells showed that threonine catabolism contributes one carbon to the same synthesis and histone methylation via the glycine cleavage pathway (Shyh-Chang et al., 2013). In recent years, glycine and N-acetylcysteine (GlyNAC) supplementation for 16 or 24 weeks has been shown to improve GSH deficiency, oxidative stress, mitochondrial dysfunction, inflammatory response, insulin resistance, muscle strength, and cognition in elderly subjects, whereas these beneficial effects were reduced 12 weeks after cessation of GlyNAC supplementation (Kumar et al., 2021; Kumar et al., 2023). A study in *C. elegans* showed that the addition of 5–500 μM glycine was able to increase the lifespan of nematode, whereas higher levels of glycine (5–10 mM) had no such effect (Liu et al., 2019). Taken together, these results suggest that glycine may serve as an aging marker.

Leucine, isoleucine, and valine all contain a branched chain structure, so they are collectively called branched-chain amino acids (BCAAs), and they are all essential amino acids. BCAAs have three main roles: as a raw material for protein synthesis, as a signaling molecule to stimulate protein synthesis, and to break down energy production during fasting (Le Couteur et al., 2020). Metabolism of BCAAs is concentrated in the muscle, and muscle tend to suffer from loss of quality and strength as the body ages. Reduced muscle anabolism and decreased response to insulin and amino acids in aging skeletal muscle, but still responsive to the administration of BCAAs, particularly leucine (Fujita and Volpi, 2006). Leucine can stimulate insulin secretion from beta cells in the pancreatic islets and work synergistically with glucose to regulate insulin secretion in response to dietary carbohydrate and protein intake (Neinast et al., 2019; Le Couteur et al., 2020). Studies have shown that a diet high in leucine can reverse the phenomenon of impaired muscle protein synthesis in the elderly, but the same diet had no significant effect on muscle protein synthesis in young adults (Katsanos et al., 2006). In addition, a study showed that skeletal muscle mass increased in the intervention group after dietary intervention with BCAA-rich supplements in patients with gait disorders, although there was no significant impact on daily activated (Moriwaki et al., 2019). However, it remains to be confirmed whether supplemental leucine can help older adults regain lost muscle mass. A systematic review and meta-analysis of leucine supplementation in older adults showed that leucine increased the rate of protein synthesis, but had no effect on lean body mass or lean leg mass in older adults (Xu et al., 2015). In addition, BCAAs levels tend to increase with age during the aging process (Table 1), and energy restriction, protein restriction, gastric bypass surgery, etc., which are thought to improve metabolism, will decrease plasma BCAA levels (Magkos et al., 2013; Zheng et al., 2016a; Fontana et al., 2016). A study examining the effect of a low-BCAAs diet on normal lifespan in mice found that starting a low-BCAAs diet midway through life increased lifespan and that this effect was associated with decreased mTORC1 signaling, while increased levels of BCAAs in the brain may increase mTORC1 signaling (Richardson et al., 2021). In addition to rodents and humans, studies of the effect of BCAAs on lifespan have been conducted in other biological models, such as *Saccharomyces cerevisiae*, *Caenorhabditis elegans*, and *Drosophila*, but the results have been inconsistent (Aris et al., 2013; Mansfeld et al., 2015; Juricic et al., 2020). In addition, BCAAs have been implicated in disease. Studies have shown that complete deprivation of BCAAs for a short period of time can improve insulin sensitivity in the liver (Xiao et al., 2011; Xiao et al., 2014). Experiments in mice and rats have shown that artificially increasing BCAA levels in the daily diet can induce obesity and insulin resistance (Newgard et al., 2009). These findings all indicate that low levels of BCAAs are beneficial for maintaining health and slowing down aging. However, plasma levels of BCAAs were lower in sarcopenic elderly subjects than in healthy elderly subjects, which means that sarcopenia is associated with reduced levels of BCAAs (Ottestad et al., 2018; Ter Borg et al., 2019). Taken together, differential changes in the levels of BCAAs in different age-related diseases suggest that BCAAs along may not be directly associated with the development of these diseases. Some scholars have noted that it may not be the right choice to group the three BCAAs into one category. The functions of leucine, valine, and isoleucine in the body are still different. For example, the blood level of isoleucine is positively correlated with human mortality, while valine and leucine show the opposite correlation (Deelen et al., 2019). Furthermore, leucine and isoleucine are insulinotropic in the postprandial phase, whereas valine and isoleucine are gluconeogenic in the fasting state (Le Couteur et al., 2020). Overall, although BCAAs may have an intriguing relationship with aging, more research is needed to further elucidate their role in aging and age-related diseases, as well as the differences between different species (Le Couteur et al., 2020; Babygirija and Lamming, 2021).

The involvement of amino acids and their derived metabolites in multiple metabolic pathways, combined with their diversity, makes it more difficult to delineate changes in their levels in aging organs from a holistic perspective. Significant changes in the levels of glutamate, glutamine, and BCAA have been reported in many publications, suggesting a close relationship between them and organ aging and making them important age-related metabolites. But their changing trends in different organs are not the same, more studies are needed to help better understand the role they may play in aging. Nevertheless, GSH or GSH/GSSG, as well as glycine levels, were decreased with aging in several organs, thus may serve as markers of aging.

### ATP and uracil in nucleotide metabolites can be used as aging markers

**FIGURE 3:**
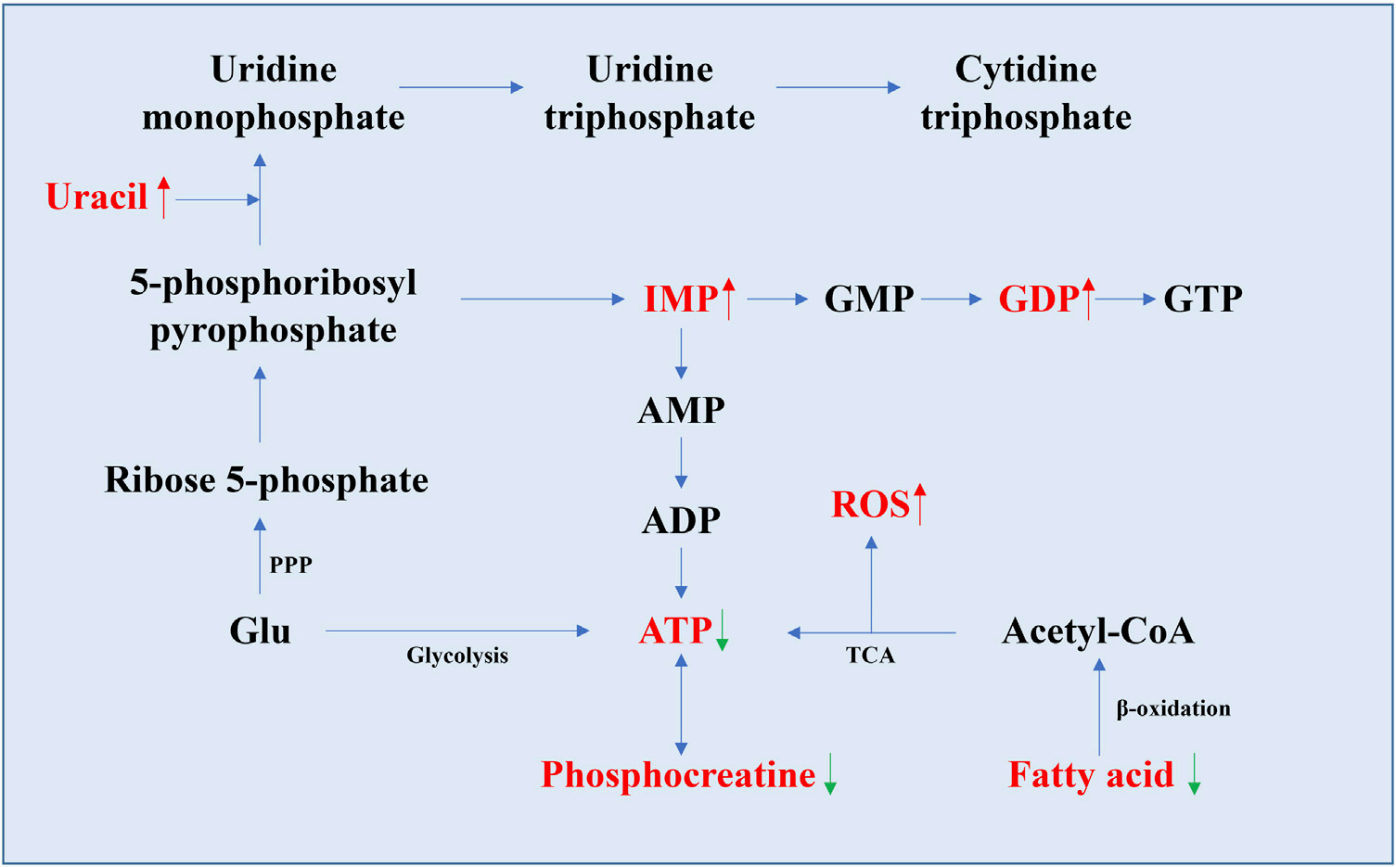
Schematic diagram of the nucleotide synthesis pathway and the pathway related to ATP generation. Metabolites with significant changes in their levels in aging organs are indicated in red font (data from Table 1). The rising trend is indicated by the red up arrow, and the decreasing trend is indicated by the green down arrow. The levels of IMP, GDP, and uracil were all relatively increased in aging organs, as well as ROS, while the levels of ATP, fatty acids, and phosphocreatine changed inversely.

Nucleotides, as the basic structural units of nucleic acids, are involved in almost all metabolic pathways in cells, such as coenzyme function, regulation, substrate activation, anabolism, and provision of nucleic acid subunits (Lane and Fan, 2015). Studies have shown that adenosine triphosphate (ATP) levels were significantly lower in the aging group in various organs of rats, mice, and human cell aging models (Table 1). The levels of inosine monophosphate (IMP), guanosine diphosphate (GDP), hypoxanthine, and uracil all increase with age (Figure 3). In the human fibroblast model, higher levels of purines (hypoxanthine, 7-methylguanine, and urate) and pyrimidine (3-ureidopropionate) and lower levels of thymidine were measured in normal and γ-ray-induced aging groups (James et al., 2015). In addition, the changes in inosine and uridine levels found in several studies varied. In the brain, heart, kidney, and liver of mice, the levels of inosine and uridine levels decreased with age (Zhang et al., 2021), whereas a study in rat showed an increase in uridine levels in the liver (Wesley et al., 2019). Increased levels of guanosine monophosphate (GMP) can be detected in the masseter muscle of aged rats (Morrison et al., 2019; Kato et al., 2021). Other substances with age-related changes in metabolite levels include deoxyribose and methylated nucleotides, which increase with age in the retina and optic nerve of aging mice, respectively (Wang et al., 2018).

The decrease in ATP is consistent with previous findings of decreased TCA cycle and OXPHOS flux in the mitochondria. Because of its important basic function in the body, it is difficult to confirm its role in the aging process. However, the total ATP level is detectable, so it is possible to make ATP a simple marker of aging with a perfect evaluation system. Hypoxanthine levels were positively correlated with age. Hypoxanthine is the product of the nucleotide degradation pathway and produces xanthine and uric acid under the action of xanthine oxidase (Lawal and Adeloju, 2012). Uracil is mentioned in several articles, and all point to a relative increase in uracil levels with age (Morrison et al., 2019; Kato et al., 2021; Zhang et al., 2021). It is a component of RNA in the form of uracil ribonucleotides, and appears in DNA in some special cases, such as abnormal cytosine deamination, and incorrect insertion of deoxy uracil dUTP during DNA synthesis (Fadda and Pomes, 2011; Lewis et al., 2016; Chakraborty and Stover, 2020). In general, uracil misincorporation into DNA is recognized and removed by DNA repair enzymes, but excess uracil mutations can cause DNA double-strand breaks, which in turn activate the p53-mediated apoptosis pathway (Yadav et al., 2016). From this perspective, the increase or accumulation of uracil that occurs during normal aging reflects the accumulation of DNA damage in organs and can be used as an aging marker.

### Lysophosphatidylcholine (LPC) and fatty acids are possible aging markers in lipid metabolism

Lipids have important functions in living organisms, including energy storage, organ protection, and the formation of cell membrane systems in living organisms. In addition, many lipid metabolites and their derivatives play key roles in cell signaling, metabolic regulation, and other processes. In aged rats, studies have shown that the levels of several glycerophospholipids (GPLs) decreased in the soleus, while glycerol, 3-phosphoglycerol, and glycerophosphocholine increased in the gastrocnemius increased (Garvey et al., 2014). Fatty acids levels of in the soleus decrease with age but increase in the gastrocnemius muscle (Garvey et al., 2014). Lower levels of carnitine and low molecular weight acylcarnitine were found in the gastrocnemius and soleus (Garvey et al., 2014). Glycerol-3-phosphate and linolenic acid levels were reduced in the liver (Son et al., 2012). However, increased levels of cholesterol, betaine, carnitine, and acylamino bases were found in the liver (Son et al., 2012). An increase in total LPC and fatty acids was found in the brain (Zheng et al., 2016b), and the same occurred in the human vastus lateralis (Zheng et al., 2016b; Wilkinson et al., 2020; Zhang et al., 2021). In addition, except for alpha-linolenic acid, the levels of omega-3 and omega-6 fatty acids levels were significantly lower, whereas linoleic acid and arachidonic acid levels were higher in the rat brain (Zheng et al., 2016b). In aged mice, fatty acids accumulate in muscle, with increased levels of polyunsaturated fatty acids and decreased levels of phospholipids such as renal phospholipids and LPC in the heart (Houtkooper et al., 2011; Zhang et al., 2021). Relatively low levels of cholesterol are found in the cornea and optic nerve (Wang et al., 2018). Glycerol decreased in kidney and liver (Eum et al., 2020; Zhang et al., 2021), but increased in the retina (Wang et al., 2018). In addition, glycerol levels were decreased, and palmitic acid, oleic acid, myristic acid, stearic acid, and linoleic acid were increased after CR treatment (Mitchell et al., 2016).

Under normal conditions, the mitochondrial membrane is polyunsaturated, and its lipid composition is dominated by unsaturated lipids. Previous studies have shown that the unsaturation of the mitochondrial membrane decreases with age (Almaida-Pagan et al., 2012; Stanley et al., 2012), resulting in reduced mitochondrial production of energy production and increased levels of ROS, causing excessive oxidative stress that can damage organs during aging (Kubota et al., 2016; Wang et al., 2018). One study showed that individuals with higher levels of LPC had greater mitochondrial oxidative capacity (Semba et al., 2019), suggesting that LPC may affect mitochondrial oxidative capacity by participating in the formation of the normal mitochondrial membrane system. In addition, studies have shown that LPC levels can be significantly altered from normal conditions in several age-related diseases, including rheumatoid arthritis, atherosclerosis, cardiovascular disease, and AD (Newgard et al., 2009; Xiao et al., 2011; Xiao et al., 2014; Ottestad et al., 2018). Therefore, LPC, a substance involved in the composition of the mitochondrial membrane structure, can be used as a universal marker of aging. On this basis, if the effective level of LPC in the body can be artificially increased by external intervention, it may have unexpected effects on delaying the aging process.

Fatty acids showed a tendency to decrease with age in most studies. However, a significant accumulation of free fatty acids was observed in aged rat gastrocnemius muscle (Garvey et al., 2014). Considering that an important pathway for energy production from fatty acids is beta-oxidation which occurs in mitochondria (Kasumov et al., 2005; Lopaschuk et al., 2010; Houten et al., 2016), it is possible that the gastrocnemius muscle attempts to compensate for the gap in energy production through fatty acid β-oxidation capacity, but due to mitochondrial damage from aging, β-oxidation cannot proceed normally, resulting in the accumulation of free fatty acids (Garvey et al., 2014). Similar results have been found in rat livers (Son et al., 2012; Garvey et al., 2014). In the above research results, the level of carnitine, a substance that helps macromolecular fatty acids enter the mitochondrial membrane, was relatively lower in the aging stage (Table 1), which may reflect the inhibited β-oxidation pathway. In addition, fatty acids may also be involved in more complex regulatory processes, such as cognitive maintenance (Pifferi et al., 2010; Pifferi et al., 2015; Royo et al., 2018). However, some studies have shown that n-3 PUFAs have an opposite regulatory effect in the human brain (Cunnane et al., 2009). Therefore, more research is needed to confirm the overall trend of changes in the levels of these substances with age.

### Polyamine metabolism, NAD^+^ and ROS

Polyamines (PAs) are ubiquitous low molecular weights biogenic amines and classically refer to three molecules: putrescine, spermidine, and spermine. Polyamines can interact with various cellular macromolecules, such as nucleic acids, ATP, proteins, and phospholipids, and thus play a critical role in many biological processes, including nucleic acids stabilization, protein synthesis, cell cycle progression, and cell proliferation (Bae et al., 2018; Igarashi and Kashiwagi, 2019). The changing trend of spermidine has different characteristics in different organs, with increasing levels in the masseter muscle of aging rats, but no significant changes levels in the colon cancer cell model (Kato et al., 2021). In contrast, spermine shows an increasing trend with aging in various organs (Wilkinson et al., 2020; Nagineni et al., 2021). Although rarely reported in the literature, polyamines may have unexpected anti-aging effects (Xu et al., 2020; Soda, 2022). One study showed that when rats were treated with spermine or spermidine, their cardiac creatine levels were reduced during aging, along with taurine, which were the same results as in CR-treated experiments (Mitchell et al., 2016; Zhang et al., 2017).

NAD^+^, NADH, nicotinamide adenine dinucleotide phosphate (NADP) and reduced nicotinamide adenine dinucleotide phosphate (NADPH), as proton transfer carriers, are responsible for the function of transferring protons in various metabolic pathways. A study in rats showed that liver NAD^+^/NADH levels decreased with age, and nicotinamide levels were also reduced (Son et al., 2012; Wesley et al., 2019). Correspondingly, NAD^+^ levels in the gastrocnemius muscle decreased significantly, consistent with the concomitant observation of glycolysis (Garvey et al., 2014). In the brain, a relative decrease in NAD^+^ levels was observed along with an increase in niacin levels (Wesley et al., 2019). Some studies have shown that lower levels of nicotinamide in the kidney, liver, and lungs of aging mice and relatively high levels of NAD^+^, riboflavin, and nicotinamide in the choroid of the eye (Wang et al., 2018; Zhang et al., 2021). In cell models, relatively low levels of acetyl-CoA were detected in senescent breast cancer cells, and the amount of NADPH produced by the PPP pathway increased in the cells of the aging and apoptosis groups, but the total amount of NADPH in the cells decreased at the same time (Wu et al., 2017). Relative decreases in nicotinamide riboside and nicotinamide ribonucleotide levels were detected in the mesenchymal stromal cell model (Fernandez-Rebollo et al., 2020), and relatively low levels of NAD^+^ and NADPH were detected in human umbilical vein endothelial cells and colon cancer cells, respectively (Yi et al., 2020; Nagineni et al., 2021). Overall, NAD^+^ shows a relative decrease in levels in aging organs, and can be used as an aging marker. Research has shown that the decrease in tissue NAD^+^ levels during aging is directly related to the increased expression of macrophage CD38^+^, a transmembrane protein that can consume NAD^+^ to form cyclic adenosine diphosphate (ADP)-ribose, ADP-ribose, and nicotinamide (Camacho-Pereira et al., 2016; Covarrubias et al., 2020).

As reported, ROS has been widely used as an aging marker. ROS are partially reduced or excited forms of oxygen, negative changes in the external environment can directly cause ROS accumulation. And cells also continue to generate endogenous ROS during normal metabolism (Sarniak et al., 2016). It is generally believed that the latter is the main reason for the marked increase in ROS levels in senescent cells. The level of intracellular ROS shows a trend of continuous accumulation throughout the life cycle of cells, and the levels of several antioxidant molecules, such as GSH, creatine, and fumarate, in senescent cells gradually decreased with increasing cell passage (Table 1), indicating that the cell’s antioxidant defense system weakens as cells age. Although the metabolomic article selected here does not provide a direct examination of ROS levels, in the free radical theory of senescence established more than five decades ago, ROS are essential for senescence and a large number of experiments to date have demonstrated the role of ROS in inducing senescence (Baranov and Baranova, 2017). However, new experimental data and clinical studies in recent years suggest that ROS also play an important role in normal cellular life processes, a finding that transforms ROS change from harmful to common metabolites like other metabolites (Baranov and Baranova, 2017; Mittler, 2017). During the development of *C. elegans*, the gradually increasing levels of ROS enhance the nematode’s ability to tolerate stress and thus have the effect of prolonging its lifespan (Bazopoulou et al., 2019). However, such findings rather emphasize the validity of ROS as a marker of senescence.

In general, the NAD^+^ levels decreased relatively in aging organs, while ROS showed a trend of continuous accumulation. Since NADPH can be used to maintain the reduced state of GSH, it is believed that this may be due to the increase in the amount of intracellular ROS, which consumes a large amount of GSH and causes the reduction of senescent cells (Wu et al., 2017). Therefore, the close relationship between ROS, NAD^+^ and aging, the fundamental role that ROS and NAD^+^ play in the life activities of cell, and the relatively mature means of detecting their levels make ROS and NAD^+^ good markers of aging.

## Conclusion

Aging, as a common phenomenon in nature, has attracted the continuous research of people for thousands of years. The study of the aging metabolome has shown that the intensity of mitochondrial TCA cycle in organs decreases during the aging stage, and at the same time, the glycolytic flux increased increases. Dysregulation of lipid oxidation pathways in normal aging and similar energy production manifestations in some age-related diseases further suggest that disruption of energy production pathways during aging may be the key to initiating or accelerating aging.

On the other hand, we also found that changes in the levels of some metabolites with the aging process have different trends in different organs, and some have obvious anteroposterior sequences, such as the gastrocnemius and soleus of rats, while others have no clear pattern in terms of current data. There may be two reasons for this phenomenon: first, the methods used were different, which in turn led to opposite results regarding changes in the levels of some metabolites. Second, it is true that different organs do not start to age at the same time. Because of their different functions, they are affected by aging to different degrees, which ultimately manifests itself as a difference in the progression of aging (Schaum et al., 2020; Tabula Muris, 2020). Since different organs often work together as a whole, combining data from multiple omics studies may be a better approach to understanding aging.

Finally, metabolomic studies directly on organs can reflect the metabolite status of these organs during aging, and the establishment of an aging organ metabolite clock may allow us to understand aging from a new perspective, provide more ways to treat aging-related diseases, and be beneficial to the health maintenance of elderly individuals. Recently, an article detailed the brains of aging and young mice, yielding a total of 1,547 different annotated metabolites (Ding et al., 2021). Through this work, the author presents a large-scale, comprehensive metabolomic map of the aging mouse brain that can inform and help researchers better understand previously established genome, transcriptome, and proteome maps (Ding et al., 2021).

Here, we use metabolomics data from aging tissues to summarize that pyruvate, GSH or GSH/GSSG levels, glycine, ATP, uracil, NAD^+^, and ROS as global aging markers that have the same change trend in different organs, while Glu, lactate, glutamate, glutamine, BCAAs, LPC, and fatty acids as potential aging markers that need more research to confirm their role in aging. We hope that this will be useful for constructing metabolomic aging clocks based on organ metabolomic data. However, at the present stage, it is extremely difficult to sample and detect organs *in vivo* compared to blood or other body fluids. In addition, some low-abundance or structurally specific small-molecule metabolites may require multiple detection methods to be effectively detected. These factors have a certain hindering effect on the promotion of metabolomic research results in organs, which may be the reason why there is no clear and accurate aging clock based on organ metabolomics to date (Rutledge et al., 2022). In conclusion, metabolomics studies of aging organs using omics techniques can truly reflect the changes we go through with aging, and will expand our understanding of aging as well as age-related diseases.

## References

[B1] Adav S. S. Wang Y. (2021). Metabolomics signatures of aging: Recent advances. Aging Dis. 12 (2), 646–661. 10.14336/AD.2020.0909 33815888 PMC7990359

[B2] Adeva-Andany M. Lopez-Ojen M. Funcasta-Calderon R. Ameneiros-Rodriguez E. Donapetry-Garcia C. Vila-Altesor M. (2014). Comprehensive review on lactate metabolism in human health. Mitochondrion 17, 76–100. 10.1016/j.mito.2014.05.007 24929216

[B3] Almaida-Pagan P. F. de Costa J. Mendiola P. Tocher D. R. (2012). Changes in tissue and mitochondrial membrane composition during rapid growth, maturation and aging in rainbow trout, oncorhynchus mykiss. Comp. Biochem. Physiol. B Biochem. Mol. Biol. 161 (4), 404–412. 10.1016/j.cbpb.2012.01.006 22281523

[B4] Anastasiou D. Poulogiannis G. Asara J. M. Boxer M. B. Jiang J. K. Shen M. (2011). Inhibition of pyruvate kinase M2 by reactive oxygen species contributes to cellular antioxidant responses. Science 334 (6060), 1278–1283. 10.1126/science.1211485 22052977 PMC3471535

[B5] Andersen J. V. Markussen K. H. Jakobsen E. Schousboe A. Waagepetersen H. S. Rosenberg P. A. (2021). Glutamate metabolism and recycling at the excitatory synapse in health and neurodegeneration. Neuropharmacology 196, 108719. 10.1016/j.neuropharm.2021.108719 34273389

[B6] Aris J. P. Alvers A. L. Ferraiuolo R. A. Fishwick L. K. Hanvivatpong A. Hu D. (2013). Autophagy and leucine promote chronological longevity and respiration proficiency during calorie restriction in yeast. Exp. Gerontol. 48 (10), 1107–1119. 10.1016/j.exger.2013.01.006 23337777 PMC3728276

[B7] Babygirija R. Lamming D. W. (2021). The regulation of healthspan and lifespan by dietary amino acids. Transl. Med. Aging 5, 17–30. 10.1016/j.tma.2021.05.001 34263088 PMC8277109

[B8] Bae D. H. Lane D. J. R. Jansson P. J. Richardson D. R. (2018). The old and new biochemistry of polyamines. Biochim. Biophys. Acta Gen. Subj. 1862 (9), 2053–2068. 10.1016/j.bbagen.2018.06.004 29890242

[B9] Baranov V. S. Baranova E. V. (2017). Aging and ambiguous ROS: System genetics analysis. Curr. aging Sci. 10 (1), 6–11. 10.2174/1874609809666160921114504 27659263

[B10] Bazopoulou D. Knoefler D. Zheng Y. Ulrich K. Oleson B. J. Xie L. (2019). Developmental ROS individualizes organismal stress resistance and lifespan. Nature 576 (7786), 301–305. 10.1038/s41586-019-1814-y 31801997 PMC7039399

[B11] Bergersen L. H. (2007). Is lactate food for neurons? Comparison of monocarboxylate transporter subtypes in brain and muscle. Neuroscience 145 (1), 11–19. 10.1016/j.neuroscience.2006.11.062 17218064

[B12] Bergman B. C. Horning M. A. Casazza G. A. Wolfel E. E. Butterfield G. E. Brooks G. A. (2000). Endurance training increases gluconeogenesis during rest and exercise in men. Am. J. Physiol. Endocrinol. Metab. 278 (2), E244–E251. 10.1152/ajpendo.2000.278.2.E244 10662708

[B13] Bouzat P. Sala N. Suys T. Zerlauth J. B. Marques-Vidal P. Feihl F. (2014). Cerebral metabolic effects of exogenous lactate supplementation on the injured human brain. Intensive Care Med. 40 (3), 412–421. 10.1007/s00134-013-3203-6 24477453

[B14] Bozzo L. Puyal J. Chatton J. Y. (2013). Lactate modulates the activity of primary cortical neurons through a receptor-mediated pathway. PLoS One 8 (8), e71721. 10.1371/journal.pone.0071721 23951229 PMC3741165

[B15] Brooks G. A. (1986). The lactate shuttle during exercise and recovery. Med. Sci. Sports Exerc 18 (3), 360–368. 10.1249/00005768-198606000-00019 3523107

[B16] Burger P. M. Mehl E. Cameron P. L. Maycox P. R. Baumert M. Lottspeich F. (1989). Synaptic vesicles immunoisolated from rat cerebral cortex contain high levels of glutamate. Neuron 3 (6), 715–720. 10.1016/0896-6273(89)90240-7 2577130

[B17] Cabreiro F. Au C. Leung K. Y. Vergara-Irigaray N. Cochemé H. M. Noori T. (2013). Metformin retards aging in *C. elegans* by altering microbial folate and methionine metabolism. Cell 153 (1), 228–239. 10.1016/j.cell.2013.02.035 23540700 PMC3898468

[B18] Camacho-Pereira J. Tarrago M. G. Chini C. C. S. Nin V. Escande C. Warner G. M. (2016). CD38 dictates age-related nad decline and mitochondrial dysfunction through an SIRT3-dependent mechanism. Cell metab. 23 (6), 1127–1139. 10.1016/j.cmet.2016.05.006 27304511 PMC4911708

[B19] Canevelli M. Lucchini F. Quarata F. Bruno G. Cesari M. (2016). Nutrition and dementia: Evidence for preventive approaches? Nutrients 8 (3), 144. 10.3390/nu8030144 26959055 PMC4808873

[B20] Cantor J. R. Sabatini D. M. (2012). Cancer cell metabolism: One hallmark, many faces. Cancer Discov. 2 (10), 881–898. 10.1158/2159-8290.CD-12-0345 23009760 PMC3491070

[B21] Chakraborty J. Stover P. J. (2020). Deoxyuracil in DNA in health and disease. Curr. Opin. Clin. Nutr. Metab. Care 23 (4), 247–252. 10.1097/MCO.0000000000000660 32398439 PMC7347158

[B22] Chao C. C. Shen P. W. Tzeng T. Y. Kung H. J. Tsai T. F. Wong Y. H. (2021). Human iPSC-derived neurons as a platform for deciphering the mechanisms behind brain aging. Biomedicines 9 (11), 1635. 10.3390/biomedicines9111635 34829864 PMC8615703

[B23] Choudhury D. Rong N. Ikhapoh I. Rajabian N. Tseropoulos G. Wu Y. (2022). Inhibition of glutaminolysis restores mitochondrial function in senescent stem cells. Cell Rep. 41 (9), 111744. 10.1016/j.celrep.2022.111744 36450260 PMC9809151

[B24] Clement J. Wong M. Poljak A. Sachdev P. Braidy N. (2019). The plasma NAD(+) metabolome is dysregulated in "normal" aging. Rejuvenation Res. 22 (2), 121–130. 10.1089/rej.2018.2077 30124109 PMC6482912

[B25] Covarrubias A. J. Kale A. Perrone R. Lopez-Dominguez J. A. Pisco A. O. Kasler H. G. (2020). Senescent cells promote tissue NAD(+) decline during ageing via the activation of CD38(+) macrophages. Nat. Metab. 2 (11), 1265–1283. 10.1038/s42255-020-00305-3 33199924 PMC7908681

[B26] Cunnane S. C. Plourde M. Pifferi F. Begin M. Feart C. Barberger-Gateau P. (2009). Fish, docosahexaenoic acid and Alzheimer's disease. Prog. Lipid Res. 48 (5), 239–256. 10.1016/j.plipres.2009.04.001 19362576

[B27] Cunnane S. C. Trushina E. Morland C. Prigione A. Casadesus G. Andrews Z. B. (2020). Brain energy rescue: An emerging therapeutic concept for neurodegenerative disorders of ageing. Nat. Rev. Drug Discov. 19 (9), 609–633. 10.1038/s41573-020-0072-x 32709961 PMC7948516

[B28] DeBerardinis R. J. Chandel N. S. (2016). Fundamentals of cancer metabolism. Sci. Adv. 2 (5), e1600200. 10.1126/sciadv.1600200 27386546 PMC4928883

[B29] Deelen J. Kettunen J. Fischer K. van der Spek A. Trompet S. Kastenmuller G. (2019). A metabolic profile of all-cause mortality risk identified in an observational study of 44,168 individuals. Nat. Commun. 10 (1), 3346. 10.1038/s41467-019-11311-9 31431621 PMC6702196

[B30] Ding J. Ji J. Rabow Z. Shen T. Folz J. Brydges C. R. (2021). A metabolome atlas of the aging mouse brain. Nat. Commun. 12 (1), 6021. 10.1038/s41467-021-26310-y 34654818 PMC8519999

[B31] Domingo-Orti I. Lamas-Domingo R. Ciudin A. Hernandez C. Herance J. R. Palomino-Schatzlein M. (2021). Metabolic footprint of aging and obesity in red blood cells. Aging (Albany NY) 13 (4), 4850–4880. 10.18632/aging.202693 33609087 PMC7950240

[B32] dos Santos J. M. Benite-Ribeiro S. A. Queiroz G. Duarte J. A. (2012). The effect of age on glucose uptake and GLUT1 and GLUT4 expression in rat skeletal muscle. Cell Biochem. Funct. 30 (3), 191–197. 10.1002/cbf.1834 22125125

[B33] Emhoff C. A. Messonnier L. A. Horning M. A. Fattor J. A. Carlson T. J. Brooks G. A. (2013). Gluconeogenesis and hepatic glycogenolysis during exercise at the lactate threshold. J. Appl. Physiol. 114 (3), 297–306. 10.1152/japplphysiol.01202.2012 23239870 PMC8846961

[B34] Escartin C. Won S. J. Malgorn C. Auregan G. Berman A. E. Chen P. C. (2011). Nuclear factor erythroid 2-related factor 2 facilitates neuronal glutathione synthesis by upregulating neuronal excitatory amino acid transporter 3 expression. J. Neurosci. 31 (20), 7392–7401. 10.1523/JNEUROSCI.6577-10.2011 21593323 PMC3339848

[B35] Eum J. Y. Lee J. C. Yi S. S. Kim I. Y. Seong J. K. Moon M. H. (2020). Aging-related lipidomic changes in mouse serum, kidney, and heart by nanoflow ultrahigh-performance liquid chromatography-tandem mass spectrometry. J. Chromatogr. A 1618, 460849. 10.1016/j.chroma.2020.460849 31928769

[B36] Fadda E. Pomes R. (2011). On the molecular basis of uracil recognition in DNA: Comparative study of T-A versus U-A structure, dynamics and open base pair kinetics. Nucleic Acids Res. 39 (2), 767–780. 10.1093/nar/gkq812 20876689 PMC3025553

[B37] Fernandez-Rebollo E. Franzen J. Goetzke R. Hollmann J. Ostrowska A. Oliverio M. (2020). Senescence-associated metabolomic phenotype in primary and iPSC-derived mesenchymal stromal cells. Stem Cell Rep. 14 (2), 201–209. 10.1016/j.stemcr.2019.12.012 PMC7013233 31983656

[B38] Ferreira A. V. Koeken V. Matzaraki V. Kostidis S. Alarcon-Barrera J. C. de Bree L. C. J. (2021). Glutathione metabolism contributes to the induction of trained immunity. Cells 10 (5), 971. 10.3390/cells10050971 33919212 PMC8143087

[B39] Flaherty D. C. Hoxha B. Sun J. Gurji H. Simecka J. W. Mallet R. T. (2010). Pyruvate-fortified fluid resuscitation improves hemodynamic stability while suppressing systemic inflammation and myocardial oxidative stress after hemorrhagic shock. Mil. Med. 175 (3), 166–172. 10.7205/milmed-d-09-00161 20358705

[B40] Fleischer J. G. Schulte R. Tsai H. H. Tyagi S. Ibarra A. Shokhirev M. N. (2018). Predicting age from the transcriptome of human dermal fibroblasts. Genome Biol. 19 (1), 221. 10.1186/s13059-018-1599-6 30567591 PMC6300908

[B41] Fontana L. Cummings N. E. Arriola Apelo S. I. Neuman J. C. Kasza I. Schmidt B. A. (2016). Decreased consumption of branched-chain amino acids improves metabolic health. Cell Rep. 16 (2), 520–530. 10.1016/j.celrep.2016.05.092 27346343 PMC4947548

[B42] Fujita S. Volpi E. (2006). Amino acids and muscle loss with aging. J. Nutr. 136, 277S–280S. 10.1093/jn/136.1.277S 16365098 PMC3183816

[B43] Garvey S. M. Dugle J. E. Kennedy A. D. McDunn J. E. Kline W. Guo L. (2014). Metabolomic profiling reveals severe skeletal muscle group-specific perturbations of metabolism in aged FBN rats. Biogerontology 15 (3), 217–232. 10.1007/s10522-014-9492-5 24652515 PMC4019835

[B44] Gaspar R. C. Munoz V. R. Nakandakari S. Vieira R. F. L. da Conceicao L. R. de Oliveira F. (2020). Aging is associated with increased TRB3, ER stress, and hepatic glucose production in the liver of rats. Exp. Gerontol. 139, 111021. 10.1016/j.exger.2020.111021 32659331

[B45] Gewirtz D. A. (1999). A critical evaluation of the mechanisms of action proposed for the antitumor effects of the anthracycline antibiotics adriamycin and daunorubicin. Biochem. Pharmacol. 57 (7), 727–741. 10.1016/s0006-2952(98)00307-4 10075079

[B46] Gruning N. M. Rinnerthaler M. Bluemlein K. Mulleder M. Wamelink M. M. Lehrach H. (2011). Pyruvate kinase triggers a metabolic feedback loop that controls redox metabolism in respiring cells. Cell metab. 14 (3), 415–427. 10.1016/j.cmet.2011.06.017 21907146 PMC3202625

[B47] Gueli M. C. Taibi G. (2013). Alzheimer's disease: Amino acid levels and brain metabolic status. Neurol. Sci. 34 (9), 1575–1579. 10.1007/s10072-013-1289-9 23354600

[B48] Hansen M. Hsu A. L. Dillin A. Kenyon C. (2005). New genes tied to endocrine, metabolic, and dietary regulation of lifespan from a *Caenorhabditis elegans* genomic RNAi screen. PLoS Genet. 1 (1), 119–128. 10.1371/journal.pgen.0010017 16103914 PMC1183531

[B49] Heilbronn L. K. Ravussin E. (2003). Calorie restriction and aging: Review of the literature and implications for studies in humans. Am. J. Clin. Nutr. 78 (3), 361–369. 10.1093/ajcn/78.3.361 12936916

[B50] Hertel J. Friedrich N. Wittfeld K. Pietzner M. Budde K. Van der Auwera S. (2016). Measuring biological age via metabonomics: The metabolic age score. J. Proteome Res. 15 (2), 400–410. 10.1021/acs.jproteome.5b00561 26652958

[B51] Homma T. Fujii J. (2015). Application of glutathione as anti-oxidative and anti-aging drugs. Curr. Drug Metab. 16 (7), 560–571. 10.2174/1389200216666151015114515 26467067

[B52] Hopkins F. G. (1929). On glutathione: A reinvestigation. J. Biol. Chem. 84 (1), 269–320. 10.1016/s0021-9258(18)77062-2

[B53] Horvath S. Raj K. (2018). DNA methylation-based biomarkers and the epigenetic clock theory of ageing. Nat. Rev. Genet. 19 (6), 371–384. 10.1038/s41576-018-0004-3 29643443

[B54] Hoshino T. Kato Y. Sugahara K. Katakura A. (2022). Aging-related metabolic changes in the extensor digitorum longus muscle of senescence-accelerated mouse-prone 8. Geriatr. Gerontol. Int. 22 (2), 160–167. 10.1111/ggi.14333 34936182 PMC9302128

[B55] Houten S. M. Violante S. Ventura F. V. Wanders R. J. (2016). The biochemistry and physiology of mitochondrial fatty acid beta-oxidation and its genetic disorders. Annu. Rev. physiology 78, 23–44. 10.1146/annurev-physiol-021115-105045 26474213

[B56] Houtkooper R. H. Argmann C. Houten S. M. Canto C. Jeninga E. H. Andreux P. A. (2011). The metabolic footprint of aging in mice. Sci. Rep. 1, 134. 10.1038/srep00134 22355651 PMC3216615

[B57] Hu S. Bai X. D. Liu X. Q. Wang H. B. Zhong Y. X. Fang T. (2013). Pyruvate ringer's solution corrects lactic acidosis and prolongs survival during hemorrhagic shock in rats. J. Emerg. Med. 45 (6), 885–893. 10.1016/j.jemermed.2013.04.062 24054887

[B58] Hunsberger H. C. Greenwood B. P. Tolstikov V. Narain N. R. Kiebish M. A. Denny C. A. (2020). Divergence in the metabolome between natural aging and Alzheimer's disease. Sci. Rep. 10 (1), 12171. 10.1038/s41598-020-68739-z 32699218 PMC7376199

[B59] Iannetti E. F. Smeitink J. A. M. Willems P. Beyrath J. Koopman W. J. H. (2018). Rescue from galactose-induced death of leigh syndrome patient cells by pyruvate and NAD. Cell Death Dis. 9 (11), 1135. 10.1038/s41419-018-1179-4 30429455 PMC6235972

[B60] Igarashi K. Kashiwagi K. (2019). The functional role of polyamines in eukaryotic cells. Int. J. Biochem. Cell Biol. 107, 104–115. 10.1016/j.biocel.2018.12.012 30578954

[B61] Inoshita M. Umehara H. Watanabe S. Y. Nakataki M. Kinoshita M. Tomioka Y. (2018). Elevated peripheral blood glutamate levels in major depressive disorder. Neuropsychiatr. Dis. Treat. 14, 945–953. 10.2147/NDT.S159855 29670355 PMC5896673

[B62] James E. L. Michalek R. D. Pitiyage G. N. de Castro A. M. Vignola K. S. Jones J. (2015). Senescent human fibroblasts show increased glycolysis and redox homeostasis with extracellular metabolomes that overlap with those of irreparable DNA damage, aging, and disease. J. Proteome Res. 14 (4), 1854–1871. 10.1021/pr501221g 25690941

[B63] Jana A. Joseph M. M. Munan S. Maiti K. K. Samanta A. (2021). A single benzene fluorescent probe for efficient formaldehyde sensing in living cells using glutathione as an amplifier. J. Photochem Photobiol. B 214, 112091. 10.1016/j.jphotobiol.2020.112091 33285487

[B64] Jarak I. Almeida S. Carvalho R. A. Sousa M. Barros A. Alves M. G. (2018). Senescence and declining reproductive potential: Insight into molecular mechanisms through testicular metabolomics. Biochim. Biophys. Acta Mol. Basis Dis. 1864 (10), 3388–3396. 10.1016/j.bbadis.2018.07.028 30059728

[B65] Johmura Y. Yamanaka T. Omori S. Wang T. W. Sugiura Y. Matsumoto M. (2021). Senolysis by glutaminolysis inhibition ameliorates various age-associated disorders. Science 371 (6526), 265–270. 10.1126/science.abb5916 33446552

[B66] Johnson L. C. Parker K. Aguirre B. F. Nemkov T. G. D'Alessandro A. Johnson S. A. (2019). The plasma metabolome as a predictor of biological aging in humans. Geroscience 41 (6), 895–906. 10.1007/s11357-019-00123-w 31707594 PMC6925078

[B67] Julio-Pieper M. Flor P. J. Dinan T. G. Cryan J. F. (2011). Exciting times beyond the brain: Metabotropic glutamate receptors in peripheral and non-neural tissues. Pharmacol. Rev. 63 (1), 35–58. 10.1124/pr.110.004036 21228260

[B68] Juricic P. Grönke S. Partridge L. (2020). Branched-chain amino acids have equivalent effects to other essential amino acids on lifespan and aging-related traits in *Drosophila* . journals gerontology Ser. A, Biol. Sci. Med. Sci. 75 (1), 24–31. 10.1093/gerona/glz080 PMC6909895 30891588

[B69] Justice J. N. Nambiar A. M. Tchkonia T. LeBrasseur N. K. Pascual R. Hashmi S. K. (2019). Senolytics in idiopathic pulmonary fibrosis: Results from a first-in-human, open-label, pilot study. EBioMedicine 40, 554–563. 10.1016/j.ebiom.2018.12.052 30616998 PMC6412088

[B70] Karakelides H. Irving B. A. Short K. R. O'Brien P. Nair K. S. (2010). Age, obesity, and sex effects on insulin sensitivity and skeletal muscle mitochondrial function. Diabetes 59 (1), 89–97. 10.2337/db09-0591 19833885 PMC2797949

[B71] Kasumov T. Adams J. E. Bian F. David F. Thomas K. R. Jobbins K. A. (2005). Probing peroxisomal beta-oxidation and the labelling of acetyl-coa proxies with [1-(13C)]octanoate and [3-(13C)]octanoate in the perfused rat liver. Biochem. J. 389 (2), 397–401. 10.1042/BJ20050144 15773815 PMC1175117

[B72] Kato Y. Hoshino T. Ogawa Y. Sugahara K. Katakura A. (2021). Metabolome analysis of masseter muscle in senescence-accelerated mouse-prone 8 (SAMP8). Resear Sq. 2021. 10.21203/rs.3.rs-389321/v1 PMC11395484 39273631

[B73] Katsanos C. S. Kobayashi H. Sheffield-Moore M. Aarsland A. Wolfe R. R. (2006). A high proportion of leucine is required for optimal stimulation of the rate of muscle protein synthesis by essential amino acids in the elderly. Am. J. Physiol. Endocrinol. Metab. 291 (2), E381–E387. 10.1152/ajpendo.00488.2005 16507602

[B74] Kendall E. C. Mason H. L. McKenzie B. F. (1930). A study of glutathione. The structure of glutathione. J. Biol. Chem. 87 (1), 55–79. 10.1016/s0021-9258(18)76892-0

[B75] Khattri R. B. Puglise J. Ryan T. E. Walter G. A. Merritt M. E. Barton E. R. (2022). Isolated murine skeletal muscles utilize pyruvate over glucose for oxidation. Metabolomics Official J. Metabolomic Soc. 18 (12), 105. 10.1007/s11306-022-01948-x PMC9732067 36480060

[B76] Kim J. Y. Lee S. H. Bae I. H. Shin D. W. Min D. Ham M. (2018). Pyruvate protects against cellular senescence through the control of mitochondrial and lysosomal function in dermal fibroblasts. J. Invest. Dermatol 138 (12), 2522–2530. 10.1016/j.jid.2018.05.033 29959907

[B77] Kondoh H. Kameda M. Yanagida M. (2020). Whole blood metabolomics in aging research. Int. J. Mol. Sci. 22 (1), 175. 10.3390/ijms22010175 33375345 PMC7796096

[B78] Kubota M. Shui Y. B. Liu M. Bai F. Huang A. J. Ma N. (2016). Mitochondrial oxygen metabolism in primary human lens epithelial cells: Association with age, diabetes and glaucoma. Free Radic. Biol. Med. 97, 513–519. 10.1016/j.freeradbiomed.2016.07.016 27445101 PMC4996752

[B79] Kuehne A. Hildebrand J. Soehle J. Wenck H. Terstegen L. Gallinat S. (2017). An integrative metabolomics and transcriptomics study to identify metabolic alterations in aged skin of humans *in vivo* . BMC Genomics 18 (1), 169. 10.1186/s12864-017-3547-3 28201987 PMC5312537

[B80] Kumar P. Liu C. Hsu J. W. Chacko S. Minard C. Jahoor F. (2021). Glycine and N-acetylcysteine (GLyNAC) supplementation in older adults improves glutathione deficiency, oxidative stress, mitochondrial dysfunction, inflammation, insulin resistance, endothelial dysfunction, genotoxicity, muscle strength, and cognition: Results of a pilot clinical trial. Clin. Transl. Med. 11 (3), e372. 10.1002/ctm2.372 33783984 PMC8002905

[B81] Kumar P. Liu C. Suliburk J. Hsu J. W. Muthupillai R. Jahoor F. (2023). Supplementing glycine and N-acetylcysteine (GLyNAC) in older adults improves glutathione deficiency, oxidative stress, mitochondrial dysfunction, inflammation, physical function, and aging hallmarks: A randomized clinical trial. journals gerontology Ser. A, Biol. Sci. Med. Sci. 78 (1), 75–89. 10.1093/gerona/glac135 PMC9879756 35975308

[B82] Kumar P. Liu C. Suliburk J. W. Minard C. G. Muthupillai R. Chacko S. (2020). Supplementing glycine and N-acetylcysteine (GLyNAC) in aging hiv patients improves oxidative stress, mitochondrial dysfunction, inflammation, endothelial dysfunction, insulin resistance, genotoxicity, strength, and cognition: Results of an open-label clinical trial. Biomedicines 8 (10), 390. 10.3390/biomedicines8100390 33007928 PMC7601820

[B83] Lane A. N. Fan T. W. (2015). Regulation of mammalian nucleotide metabolism and biosynthesis. Nucleic Acids Res. 43 (4), 2466–2485. 10.1093/nar/gkv047 25628363 PMC4344498

[B84] Lauritzen K. H. Morland C. Puchades M. Holm-Hansen S. Hagelin E. M. Lauritzen F. (2014). Lactate receptor sites link neurotransmission, neurovascular coupling, and brain energy metabolism. Cereb. Cortex 24 (10), 2784–2795. 10.1093/cercor/bht136 23696276

[B85] Lawal A. T. Adeloju S. B. (2012). Progress and recent advances in fabrication and utilization of hypoxanthine biosensors for meat and fish quality assessment: A review. Talanta 100, 217–228. 10.1016/j.talanta.2012.07.085 23141330

[B86] Le A. Cooper C. R. Gouw A. M. Dinavahi R. Maitra A. Deck L. M. (2010). Inhibition of lactate dehydrogenase a induces oxidative stress and inhibits tumor progression. Proc. Natl. Acad. Sci. U. S. A. 107 (5), 2037–2042. 10.1073/pnas.0914433107 20133848 PMC2836706

[B87] Le Couteur D. G. Solon-Biet S. M. Cogger V. C. Ribeiro R. de Cabo R. Raubenheimer D. (2020). Branched chain amino acids, aging and age-related health. Ageing Res. Rev. 64, 101198. 10.1016/j.arr.2020.101198 33132154

[B88] Lewis C. A. Jr. Crayle J. Zhou S. Swanstrom R. Wolfenden R. (2016). Cytosine deamination and the precipitous decline of spontaneous mutation during Earth's history. Proc. Natl. Acad. Sci. U. S. A. 113 (29), 8194–8199. 10.1073/pnas.1607580113 27382162 PMC4961170

[B89] Li M. Zhou S. Chen C. Ma L. Luo D. Tian X. (2020). Therapeutic potential of pyruvate therapy for patients with mitochondrial diseases: A systematic review. Ther. Adv. Endocrinol. Metab. 11, 2042018820938240. 10.1177/2042018820938240 32695307 PMC7350055

[B90] Li W. Li M. Qi J. (2021). Nano-drug design based on the physiological properties of glutathione. Molecules 26 (18), 5567. 10.3390/molecules26185567 34577040 PMC8469141

[B91] Liu Y. J. Janssens G. E. McIntyre R. L. Molenaars M. Kamble R. Gao A. W. (2019). Glycine promotes longevity in *Caenorhabditis elegans* in a methionine cycle-dependent fashion. PLoS Genet. 15 (3), e1007633. 10.1371/journal.pgen.1007633 30845140 PMC6424468

[B92] Locasale J. W. (2013). Serine, glycine and one-carbon units: Cancer metabolism in full circle. Nat. Rev. Cancer 13 (8), 572–583. 10.1038/nrc3557 23822983 PMC3806315

[B93] Lopaschuk G. D. Ussher J. R. Folmes C. D. Jaswal J. S. Stanley W. C. (2010). Myocardial fatty acid metabolism in health and disease. Physiol. Rev. 90 (1), 207–258. 10.1152/physrev.00015.2009 20086077

[B94] Magkos F. Bradley D. Schweitzer G. G. Finck B. N. Eagon J. C. Ilkayeva O. (2013). Effect of Roux-en-Y gastric bypass and laparoscopic adjustable gastric banding on branched-chain amino acid metabolism. Diabetes 62 (8), 2757–2761. 10.2337/db13-0185 23610059 PMC3717831

[B95] Mansfeld J. Urban N. Priebe S. Groth M. Frahm C. Hartmann N. (2015). Branched-chain amino acid catabolism is a conserved regulator of physiological ageing. Nat. Commun. 6, 10043. 10.1038/ncomms10043 26620638 PMC4686672

[B96] Mattson M. P. Arumugam T. V. (2018). Hallmarks of brain aging: Adaptive and pathological modification by metabolic states. Cell metab. 27 (6), 1176–1199. 10.1016/j.cmet.2018.05.011 29874566 PMC6039826

[B97] Meyer C. Dostou J. M. Welle S. L. Gerich J. E. (2002). Role of human liver, kidney, and skeletal muscle in postprandial glucose homeostasis. Am. J. Physiol. Endocrinol. Metab. 282 (2), E419–E427. 10.1152/ajpendo.00032.2001 11788375

[B98] Mitchell J. B. Russo A. Kuppusamy P. Krishna M. C. (2000). Radiation, radicals, and images. Ann. N. Y. Acad. Sci. 899, 28–43. 10.1111/j.1749-6632.2000.tb06174.x 10863527

[B99] Mitchell S. J. Madrigal-Matute J. Scheibye-Knudsen M. Fang E. Aon M. Gonzalez-Reyes J. A. (2016). Effects of sex, strain, and energy intake on hallmarks of aging in mice. Cell metab. 23 (6), 1093–1112. 10.1016/j.cmet.2016.05.027 27304509 PMC4911707

[B100] Mittler R. (2017). ROS are good. Trends Plant Sci. 22 (1), 11–19. 10.1016/j.tplants.2016.08.002 27666517

[B101] MoriwakiM.WakabayashiH.SakataK.DomenK. (2019). The effect of branched chain amino acids-enriched nutritional supplements on activities of daily living and muscle mass in inpatients with gait impairments: A randomized controlled trial. J. Nutr. health & aging 23 (4), 348–353. 10.1007/s12603-019-1172-3 30932133

[B102] Morrison E. J. Champagne D. P. Dzieciatkowska M. Nemkov T. Zimring J. C. Hansen K. C. (2019). Parabiosis incompletely reverses aging-induced metabolic changes and oxidant stress in mouse red blood cells. Nutrients 11 (6), 1337. 10.3390/nu11061337 31207887 PMC6627295

[B103] Muzumdar R. Ma X. Atzmon G. Vuguin P. Yang X. Barzilai N. (2004). Decrease in glucose-stimulated insulin secretion with aging is independent of insulin action. Diabetes 53 (2), 441–446. 10.2337/diabetes.53.2.441 14747296

[B104] Nagineni C. N. Naz S. Choudhuri R. Chandramouli G. V. R. Krishna M. C. Brender J. R. (2021). Radiation-induced senescence reprograms secretory and metabolic pathways in colon cancer HCT-116 cells. Int. J. Mol. Sci. 22 (9), 4835. 10.3390/ijms22094835 34063570 PMC8124941

[B105] Neinast M. Murashige D. Arany Z. (2019). Branched chain amino acids. Annu. Rev. physiology 81, 139–164. 10.1146/annurev-physiol-020518-114455 PMC6536377 30485760

[B106] Newgard C. B. An J. Bain J. R. Muehlbauer M. J. Stevens R. D. Lien L. F. (2009). A branched-chain amino acid-related metabolic signature that differentiates obese and lean humans and contributes to insulin resistance. Cell metab. 9 (4), 311–326. 10.1016/j.cmet.2009.02.002 19356713 PMC3640280

[B107] Oliveira J. R. S. Mohamed J. S. Myers M. J. Brooks M. J. Alway S. E. (2019). Effects of hindlimb suspension and reloading on gastrocnemius and soleus muscle mass and function in geriatric mice. Exp. Gerontol. 115, 19–31. 10.1016/j.exger.2018.11.011 30448397 PMC6366863

[B108] Olstad E. Qu H. Sonnewald U. (2007). Glutamate is preferred over glutamine for intermediary metabolism in cultured cerebellar neurons. J. Cereb. Blood Flow. Metab. 27 (4), 811–820. 10.1038/sj.jcbfm.9600400 17033695

[B109] Ottestad I. Ulven S. M. Oyri L. K. L. Sandvei K. S. Gjevestad G. O. Bye A. (2018). Reduced plasma concentration of branched-chain amino acids in sarcopenic older subjects: A cross-sectional study. Br. J. Nutr. 120 (4), 445–453. 10.1017/S0007114518001307 29909813

[B110] Pavlova N. N. Thompson C. B. (2016). The emerging hallmarks of cancer metabolism. Cell metab. 23 (1), 27–47. 10.1016/j.cmet.2015.12.006 26771115 PMC4715268

[B111] Pifferi F. Dorieux O. Castellano C. A. Croteau E. Masson M. Guillermier M. (2015). Long-chain n-3 PUFAs from fish oil enhance resting state brain glucose utilization and reduce anxiety in an adult nonhuman primate, the grey mouse lemur. J. Lipid Res. 56 (8), 1511–1518. 10.1194/jlr.M058933 26063461 PMC4513992

[B112] Pifferi F. Jouin M. Alessandri J. M. Roux F. Perriere N. Langelier B. (2010). N-3 long-chain fatty acids and regulation of glucose transport in two models of rat brain endothelial cells. Neurochem. Int. 56 (5), 703–710. 10.1016/j.neuint.2010.02.006 20153394

[B113] Rebrin I. Sohal R. S. (2008). Pro-oxidant shift in glutathione redox state during aging. Adv. Drug Deliv. Rev. 60 (13-14), 1545–1552. 10.1016/j.addr.2008.06.001 18652861 PMC2585506

[B114] Richardson N. E. Konon E. N. Schuster H. S. Mitchell A. T. Boyle C. Rodgers A. C. (2021). Lifelong restriction of dietary branched-chain amino acids has sex-specific benefits for frailty and lifespan in mice. Nat. Aging 1 (1), 73–86. 10.1038/s43587-020-00006-2 33796866 PMC8009080

[B115] Roberts J. A. Varma V. R. Huang C. W. An Y. Oommen A. Tanaka T. (2020). Blood metabolite signature of metabolic syndrome implicates alterations in amino acid metabolism: Findings from the baltimore longitudinal study of aging (BLSA) and the tsuruoka metabolomics cohort study (TMCS). Int. J. Mol. Sci. 21 (4), 1249. 10.3390/ijms21041249 32070008 PMC7072861

[B116] Robinson O. Chadeau Hyam M. Karaman I. Climaco Pinto R. Ala-Korpela M. Handakas E. (2020). Determinants of accelerated metabolomic and epigenetic aging in a UK cohort. Aging Cell 19 (6), e13149. 10.1111/acel.13149 32363781 PMC7294785

[B117] Royo J. Villain N. Champeval D. Del Gallo F. Bertini G. Aujard F. (2018). Effects of n-3 polyunsaturated fatty acid supplementation on cognitive functions, electrocortical activity and neurogenesis in a non-human primate, the grey mouse lemur (*Microcebus murinus*). Behav. Brain Res. 347, 394–407. 10.1016/j.bbr.2018.02.029 29486268

[B118] Rupsingh R. Borrie M. Smith M. Wells J. L. Bartha R. (2011). Reduced hippocampal glutamate in Alzheimer disease. Neurobiol. Aging 32 (5), 802–810. 10.1016/j.neurobiolaging.2009.05.002 19501936

[B119] Rutledge J. Oh H. Wyss-Coray T. (2022). Measuring biological age using omics data. Nat. Rev. Genet. 23 (12), 715–727. 10.1038/s41576-022-00511-7 35715611 PMC10048602

[B120] Sanders C. Behrens S. Schwartz S. Wengreen H. Corcoran C. D. Lyketsos C. G. (2016). Nutritional status is associated with faster cognitive decline and worse functional impairment in the progression of dementia: The cache county dementia progression study1. J. Alzheimers Dis. 52 (1), 33–42. 10.3233/JAD-150528 26967207 PMC5318140

[B121] Sarniak A. Lipinska J. Tytman K. Lipinska S. (2016). Endogenous mechanisms of reactive oxygen species (ROS) generation. Postepy Hig. Med. Dosw (Online). 70 (0), 1150–1165. 10.5604/17322693.1224259 27892899

[B122] Schaum N. Lehallier B. Hahn O. Palovics R. Hosseinzadeh S. Lee S. E. (2020). Ageing hallmarks exhibit organ-specific temporal signatures. Nature 583 (7817), 596–602. 10.1038/s41586-020-2499-y 32669715 PMC7757734

[B123] Scire A. Cianfruglia L. Minnelli C. Bartolini D. Torquato P. Principato G. (2019). Glutathione compartmentalization and its role in glutathionylation and other regulatory processes of cellular pathways. Biofactors 45 (2), 152–168. 10.1002/biof.1476 30561781

[B124] Semba R. D. Zhang P. Adelnia F. Sun K. Gonzalez-Freire M. Salem N. Jr. (2019). Low plasma lysophosphatidylcholines are associated with impaired mitochondrial oxidative capacity in adults in the baltimore longitudinal study of aging. Aging Cell 18 (2), e12915. 10.1111/acel.12915 30719830 PMC6413748

[B125] Shao Y. Le W. (2019). Recent advances and perspectives of metabolomics-based investigations in Parkinson's disease. Mol. Neurodegener. 14 (1), 3. 10.1186/s13024-018-0304-2 30634989 PMC6330496

[B126] Shyh-Chang N. Locasale J. W. Lyssiotis C. A. Zheng Y. Teo R. Y. Ratanasirintrawoot S. (2013). Influence of threonine metabolism on S-adenosylmethionine and histone methylation. Sci. (New York, NY) 339 (6116), 222–226. 10.1126/science.1226603 PMC3652341 23118012

[B127] Sibson N. R. Dhankhar A. Mason G. F. Rothman D. L. Behar K. L. Shulman R. G. (1998). Stoichiometric coupling of brain glucose metabolism and glutamatergic neuronal activity. Proc. Natl. Acad. Sci. U. S. A. 95 (1), 316–321. 10.1073/pnas.95.1.316 9419373 PMC18211

[B128] Smith D. Pernet A. Hallett W. A. Bingham E. Marsden P. K. Amiel S. A. (2003). Lactate: A preferred fuel for human brain metabolism *in vivo* . J. Cereb. Blood Flow. Metab. 23 (6), 658–664. 10.1097/01.WCB.0000063991.19746.11 12796713

[B129] Soda K. (2022). Overview of polyamines as nutrients for human healthy long life and effect of increased polyamine intake on DNA methylation. Cells 11 (1), 164. 10.3390/cells11010164 35011727 PMC8750749

[B130] Someya S. Kim M. J. (2021). Cochlear detoxification: Role of alpha class glutathione transferases in protection against oxidative lipid damage, ototoxicity, and cochlear aging. Hear Res. 402, 108002. 10.1016/j.heares.2020.108002 32600853 PMC7704621

[B131] Son N. Hur H. J. Sung M. J. Kim M. S. Hwang J. T. Park J. H. (2012). Liquid chromatography-mass spectrometry-based metabolomic analysis of livers from aged rats. J. Proteome Res. 11 (4), 2551–2558. 10.1021/pr201263q 22380686

[B132] Sonnewald U. White L. R. Odegard E. Westergaard N. Bakken I. J. Aasly J. (1996). MRS study of glutamate metabolism in cultured neurons/glia. Neurochem. Res. 21 (9), 987–993. 10.1007/BF02532408 8897461

[B133] Stanley W. C. Khairallah R. J. Dabkowski E. R. (2012). Update on lipids and mitochondrial function: Impact of dietary n-3 polyunsaturated fatty acids. Curr. Opin. Clin. Nutr. Metab. Care 15 (2), 122–126. 10.1097/MCO.0b013e32834fdaf7 22248591 PMC4067133

[B134] Tabula Muris C. (2020). A single-cell transcriptomic atlas characterizes ageing tissues in the mouse. Nature 583 (7817), 590–595. 10.1038/s41586-020-2496-1 32669714 PMC8240505

[B135] Tanaka T. Biancotto A. Moaddel R. Moore A. Z. Gonzalez-Freire M. Aon M. A. (2018). Plasma proteomic signature of age in healthy humans. Aging Cell 17 (5), e12799. 10.1111/acel.12799 29992704 PMC6156492

[B136] Tepp K. Puurand M. Timohhina N. Adamson J. Klepinin A. Truu L. (2017). Changes in the mitochondrial function and in the efficiency of energy transfer pathways during cardiomyocyte aging. Mol. Cell Biochem. 432 (1-2), 141–158. 10.1007/s11010-017-3005-1 28293876

[B137] Ter BorgS.LuikingY. C.van HelvoortA.BoirieY.ScholsJ.de GrootC. (2019). Low levels of branched chain amino acids, eicosapentaenoic acid and micronutrients are associated with low muscle mass, strength and function in community-dwelling older adults. J. Nutr. health & aging 23 (1), 27–34. 10.1007/s12603-018-1108-3 30569065

[B138] Thorn C. F. Oshiro C. Marsh S. Hernandez-Boussard T. McLeod H. Klein T. E. (2011). Doxorubicin pathways: Pharmacodynamics and adverse effects. Pharmacogenet Genomics 21 (7), 440–446. 10.1097/FPC.0b013e32833ffb56 21048526 PMC3116111

[B139] Tremolizzo L. Sala G. Zoia C. P. Ferrarese C. (2012). Assessing glutamatergic function and dysfunction in peripheral tissues. Curr. Med. Chem. 19 (9), 1310–1315. 10.2174/092986712799462702 22304709

[B140] Tryggvason K. Wartiovaara J. (2005). How does the kidney filter plasma? Physiol. (Bethesda) 20 (2), 96–101. 10.1152/physiol.00045.2004 15772298

[B141] Uneyama H. Kobayashi H. Tonouchi N. (2017). New functions and potential applications of amino acids. Adv. Biochem. Eng. Biotechnol. 159, 273–287. 10.1007/10_2016_35 27872968

[B142] van den Akker E. B. Trompet S. Wolf Jjh B. Beekman M. Suchiman H. E. D. Deelen J. (2020). Metabolic age based on the BBMRI-NL ^1^H-NMR metabolomics repository as biomarker of age-related disease. Circ. Genom Precis. Med. 13 (5), 541–547. 10.1161/CIRCGEN.119.002610 33079603

[B143] Van PilsumJ. F.StephensG. C.TaylorD. (1972). Distribution of creatine, guanidinoacetate and the enzymes for their biosynthesis in the animal kingdom. Implications for phylogeny. Biochem. J. 126 (2), 325–345. 10.1042/bj1260325 5071177

[B144] Verdin E. (2015). NAD⁺ in aging, metabolism, and neurodegeneration. Science 350 (6265), 1208–1213. 10.1126/science.aac4854 26785480

[B145] Wang Y. Grenell A. Zhong F. Yam M. Hauer A. Gregor E. (2018). Metabolic signature of the aging eye in mice. Neurobiol. Aging 71, 223–233. 10.1016/j.neurobiolaging.2018.07.024 30172221 PMC6162115

[B146] Watkins J. C. Jane D. E. (2006). The glutamate story. Br. J. Pharmacol. 147, S100–S108. 10.1038/sj.bjp.0706444 16402093 PMC1760733

[B147] Wesley U. V. Bhute V. J. Hatcher J. F. Palecek S. P. Dempsey R. J. (2019). Local and systemic metabolic alterations in brain, plasma, and liver of rats in response to aging and ischemic stroke, as detected by nuclear magnetic resonance (NMR) spectroscopy. Neurochem. Int. 127, 113–124. 10.1016/j.neuint.2019.01.025 30707914

[B148] Wilkinson D. J. Rodriguez-Blanco G. Dunn W. B. Phillips B. E. Williams J. P. Greenhaff P. L. (2020). Untargeted metabolomics for uncovering biological markers of human skeletal muscle ageing. Aging (Albany NY) 12 (13), 12517–12533. 10.18632/aging.103513 32580166 PMC7377844

[B149] World Health Administration (2020). Decade of healthy ageing: The global strategy and action plan on ageing and health 2016–2020: Towards a world in which everyone can live a long and healthy life: Report by the director-general. Geneva: World Health Organization.

[B150] Wu M. Ye H. Shao C. Zheng X. Li Q. Wang L. (2017). Metabolomics-proteomics combined approach identifies differential metabolism-associated molecular events between senescence and apoptosis. J. Proteome Res. 16 (6), 2250–2261. 10.1021/acs.jproteome.7b00111 28467092

[B151] Xiao F. Huang Z. Li H. Yu J. Wang C. Chen S. (2011). Leucine deprivation increases hepatic insulin sensitivity via GCN2/mTOR/S6K1 and AMPK pathways. Diabetes 60 (3), 746–756. 10.2337/db10-1246 21282364 PMC3046835

[B152] Xiao F. Yu J. Guo Y. Deng J. Li K. Du Y. (2014). Effects of individual branched-chain amino acids deprivation on insulin sensitivity and glucose metabolism in mice. Metabolism 63 (6), 841–850. 10.1016/j.metabol.2014.03.006 24684822

[B153] Xu M. Pirtskhalava T. Farr J. N. Weigand B. M. Palmer A. K. Weivoda M. M. (2018). Senolytics improve physical function and increase lifespan in old age. Nat. Med. 24 (8), 1246–1256. 10.1038/s41591-018-0092-9 29988130 PMC6082705

[B154] Xu T. T. Li H. Dai Z. Lau G. K. Li B. Y. Zhu W. L. (2020). Spermidine and spermine delay brain aging by inducing autophagy in SAMP8 mice. Aging (Albany NY) 12 (7), 6401–6414. 10.18632/aging.103035 32268299 PMC7185103

[B155] Xu Z. R. Tan Z. J. Zhang Q. Gui Q. F. Yang Y. M. (2015). The effectiveness of leucine on muscle protein synthesis, lean body mass and leg lean mass accretion in older people: A systematic review and meta-analysis. Br. J. Nutr. 113 (1), 25–34. 10.1017/S0007114514002475 25234223

[B156] Yadav M. K. Manoli N. M. Madhunapantula S. V. (2016). Comparative assessment of vitamin-B12, folic acid and homocysteine levels in relation to p53 expression in megaloblastic anemia. PLoS One 11 (10), e0164559. 10.1371/journal.pone.0164559 27780269 PMC5079580

[B157] Yanar K. Simsek B. Atukeren P. Aydin S. Cakatay U. (2019). Is D-galactose a useful agent for accelerated aging model of gastrocnemius and soleus muscle of sprague-dawley rats? Rejuvenation Res. 22 (6), 521–528. 10.1089/rej.2019.2185 31131732

[B158] Yi S. Lin K. Jiang T. Shao W. Huang C. Jiang B. (2020). NMR-based metabonomic analysis of HUVEC cells during replicative senescence. Aging (Albany NY) 12 (4), 3626–3646. 10.18632/aging.102834 32074082 PMC7066908

[B159] Yu Y. Herman P. Rothman D. L. Agarwal D. Hyder F. (2018). Evaluating the gray and white matter energy budgets of human brain function. J. Cereb. Blood Flow. Metab. 38 (8), 1339–1353. 10.1177/0271678X17708691 28589753 PMC6092772

[B160] Zhang F. Kerbl-Knapp J. Akhmetshina A. Korbelius M. Kuentzel K. B. Vujic N. (2021). Tissue-specific landscape of metabolic dysregulation during ageing. Biomolecules 11 (2), 235. 10.3390/biom11020235 33562384 PMC7914945

[B161] Zhang H. Wang J. Li L. Chai N. Chen Y. Wu F. (2017). Spermine and spermidine reversed age-related cardiac deterioration in rats. Oncotarget 8 (39), 64793–64808. 10.18632/oncotarget.18334 29029392 PMC5630292

[B162] Zhang X. M. Deng H. Tong J. D. Wang Y. Z. Ning X. C. Yang X. H. (2020b). Pyruvate-enriched oral rehydration solution improves glucometabolic disorders in the kidneys of diabetic db/db mice. J. Diabetes Res. 2020, 2817972. 10.1155/2020/2817972 33062708 PMC7533008

[B163] Zhang X. M. Wang Y. Z. Tong J. D. Ning X. C. Zhou F. Q. Yang X. H. (2020a). Pyruvate alleviates high glucose-induced endoplasmic reticulum stress and apoptosis in HK-2 cells. FEBS Open Bio 10 (5), 827–834. 10.1002/2211-5463.12834 PMC7193158 32150786

[B164] Zheng X. Chen T. Zhao A. Wang X. Xie G. Huang F. (2016b). The brain metabolome of male rats across the lifespan. Sci. Rep. 6, 24125. 10.1038/srep24125 27063670 PMC4827083

[B165] Zheng Y. Ceglarek U. Huang T. Li L. Rood J. Ryan D. H. (2016a). Weight-loss diets and 2-y changes in circulating amino acids in 2 randomized intervention trials. Am. J. Clin. Nutr. 103 (2), 505–511. 10.3945/ajcn.115.117689 26791187 PMC4733257

[B166] Zhou F. Q. (2021). NAD(+), senolytics, or pyruvate for healthy aging? Nutr. metabolic insights 2021, 117863882110534. 10.1177/11786388211053407 PMC8552375 34720589

[B167] Zhou Q. Kerbl-Knapp J. Zhang F. Korbelius M. Kuentzel K. B. Vujic N. (2021). Metabolomic profiles of mouse tissues reveal an interplay between aging and energy metabolism. Metabolites 12 (1), 17. 10.3390/metabo12010017 35050139 PMC8779655

[B168] Zhou Y. Danbolt N. C. (2014). Glutamate as a neurotransmitter in the healthy brain. J. Neural Transm. (Vienna) 121 (8), 799–817. 10.1007/s00702-014-1180-8 24578174 PMC4133642

[B169] Zhuang H. Karvinen S. Tormakangas T. Zhang X. Ojanen X. Velagapudi V. (2021). Interactive effects of aging and aerobic capacity on energy metabolism-related metabolites of serum, skeletal muscle, and white adipose tissue. Geroscience 43 (6), 2679–2691. 10.1007/s11357-021-00387-1 34089174 PMC8602622

